# Corosolic acid sensitizes ferroptosis by upregulating HERPUD1 in liver cancer cells

**DOI:** 10.1038/s41420-022-01169-0

**Published:** 2022-08-29

**Authors:** Yingxiu Peng, Ning Li, Feifeng Tang, Chunmei Qian, Tingting Jia, Jingjin Liu, Yanfeng Xu

**Affiliations:** 1 Department of Pharmacy, Shanghai Municipal Hospital of Traditional Chinese Medicine, Shanghai University of Traditional Chinese Medicine, 200071 Shanghai, China; 2 Central Laboratory, Shanghai Municipal Hospital of Traditional Chinese Medicine, Shanghai University of Traditional Chinese Medicine, 200071 Shanghai, China

**Keywords:** Cancer therapy, Cell death, Pharmacology

## Abstract

Primary liver cancer is the third leading cause of cancer death in the world, and the lack of effective treatments is the main reason for the high mortality. Corosolic acid (CA) has been proved to have antitumor activity. In this study, we found that CA can sensitize liver cancer cells to ferroptosis, which is a regulated form of cell death characterized by iron-dependent lipid peroxides reaching lethal levels. Here, we revealed that CA can inhibit glutathione (GSH) synthesis via HERPUD1, decreasing the cellular GSH level and causing liver cancer cells to become more sensitive to ferroptosis. Mechanistically, further studies found that HERPUD1 reduced the ubiquitination of the GSS-associated E3 ubiquitin ligase MDM2, which promoted ubiquitination of GSS, thereby inhibiting GSH synthesis to increase ferroptosis susceptibility. Importantly, a mouse xenograft model also demonstrated that CA inhibits tumor growth via HERPUD1. Collectively, our findings suggesting that CA is a candidate component for the development of treatments against liver cancer.

## Introduction

Cancer is a major public problem in all countries of the world, and it is the leading cause of shortening lifespan and leading to death [1]. The liver is the sixth most common site of primary cancers, but liver cancer is the third leading cause of cancer-related death globally [2]. Hepatocellular carcinoma (HCC) is the most prevalent type of primary liver cancer, accounting for ~75–85% of cases [2]. Lack of effective drug treatments is one of the main reasons for the high mortality of liver cancer patients. Therefore, there is an urgent need to develop more effective HCC treatment drugs.

Ferroptosis is a type of programmed cell death that has been discovered in recent years, which is morphologically, biochemically, and genetically distinct from other forms of cell death [3–5]. The characteristic of this process is that it relies on ferrous ions and reactive oxygen species (ROS) to increase the amount of lipid peroxide (LPO) accumulation. Numerous metabolic pathways related to lipids, iron and amino acids have been reported to control cellular susceptibility to ferroptosis [6]. Recent studies have shown that ferroptosis inducers can increase lipid ROS by directly or indirectly inhibiting glutathione (GSH) synthesis and ultimately lead to cell death [7]. Liver cancer cells can be cleared by ferroptosis [8, 9]. RAS-selective lethal 3 (RSL3) and erastin can induce ferroptosis and liver cancer cell death [10, 11]. Furthermore, liver cancer may be induced by inhibiting ferroptosis [12–14], which provides a new method for fighting liver cancer in clinical practice.

**Fig. 1:**
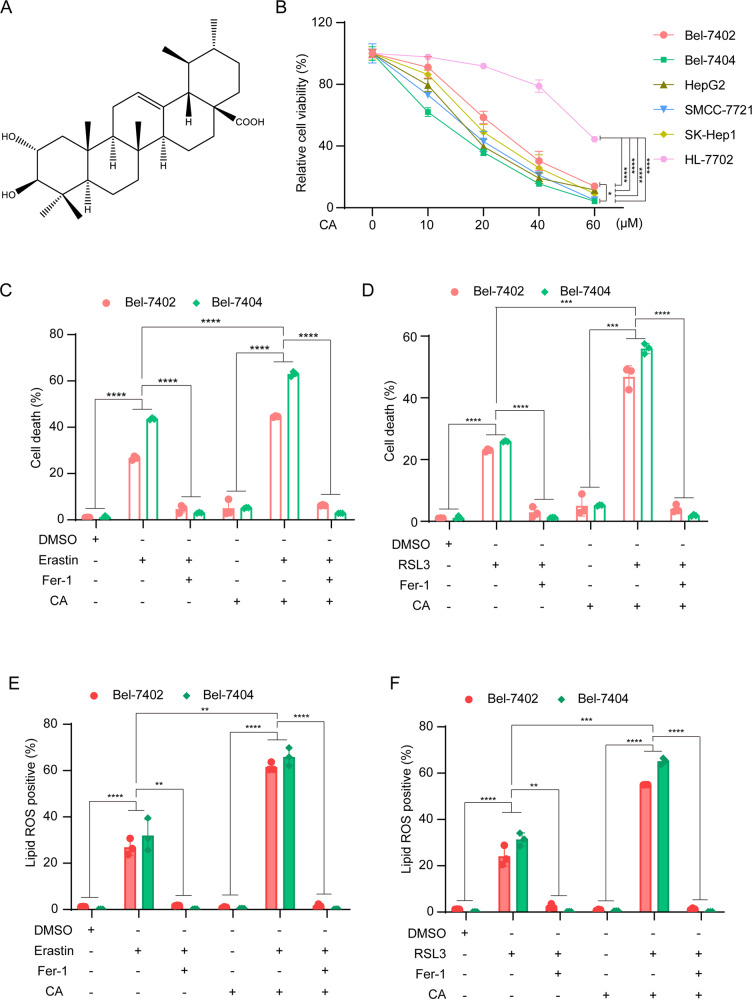
CA increases the sensitivity of liver cancer cells to ferroptosis. **A** Chemical structure of CA. **B** Established HCC cell lines (Bel-7402, Bel-7404, SMCC-7721, SK-Hep1, HepG2) and normal hepatocyte cell (HL-7702) were treated with a series of concentrations (0, 10, 20, 40, 60 μM) of CA for 24 h, and cell viability was determined by CCK-8 assay. **C**, **D** Bel-7402 and Bel-7404 cells were seeded at an appropriate density in six-well plates and cultured for 24 h, then treated with: 10 μM erastin or 5 μM RSL3; 10 μM CA; pretreatment with 10 μM CA for 1 h followed by 10 μM erastin or 5 μM RSL3; 2 μM ferroptosis inhibitor Fer-1, in complete medium for 24 h. Cell death was quantified by SYTOX-Green staining followed by flow cytometry. **E**, **F** Production of lipid ROS in (**C**, **D**) was measured by C11-BODIPY staining followed by flow cytometry after 10 h of drug treatment: 10 μM erastin or 5 μM RSL3; 10 μM CA; pretreatment with 10 μM CA for 1 h followed by 10 μM erastin or 5 μM RSL3. Data were analyzed using Student’s *t* test and expressed as mean ± SD from three independent experiments. **P < 0.01; ***P < 0.001; ****P < 0.0001.

Corosolic acid (CA; Fig. 1A) is a monomer extracted from the root of *Actinidia valvata Dunn*. As a natural pentacyclic triterpene acid, CA has been shown to have many properties, such as anti-diabetes [15], enhancing weight loss, anti-inflammatory activities [16], antiviral and anti-cardiovascular disease effects [17]. It was reported that CA has antitumor activities [18–20]. In a study of the antitumor activity of CA, it was found that CA can inhibit tumor cell cycle and induce tumor cell apoptosis [21]. In addition, CA has been proven to have pharmacological activity against cancers such as renal cancer [22], gastric cancer [23], colon cancer [24], osteosarcoma cells [25], and others. In our previous study, we further found that actinomycin D can inhibit the activity of Yes-associated protein (YAP), thereby enhancing the anti-liver cancer activity of CA [26]. There is no relevant report on the effect of CA on ferroptosis of liver cancer cells. In this study, we found for the first time that CA can sensitize liver cancer cells to ferroptosis. Furthermore, we investigated the mechanism by which CA increases the ferroptosis sensitivity of human liver cancer cells, and revealed that CA sensitized ferroptosis by upregulating Homocysteine inducible ER protein with ubiquitin-like domain 1 (HERPUD1) in liver cancer cells. Importantly, in both cellular and mouse xenograft models, we demonstrated that HERPUD1 reduced the ubiquitination of the GSS-associated E3 ubiquitin ligase MDM2, which promoted ubiquitination of GSS, thereby inhibiting GSH synthesis to increase ferroptosis susceptibility.

## Results

### Corosolic acid can increase the sensitivity of liver cancer cells to ferroptosis

In order to explore the effect of CA on the cell viability of liver cancer cells, five hepatocellular carcinoma cell lines, Bel-7402, Bel-7404, HepG2, SMCC-7721, SK-Hep1, and a normal hepatocyte cell line HL-7702 were treated with a series of concentrations of CA (0, 10, 20, 40, and 60 μM), and the CCK-8 assay was performed. Among the five liver cancer cell lines, Bel-7404 is relatively more sensitive to CA, while Bel-7402 is relatively less sensitive to CA (Fig. 1B). Interestingly, we found that CA has almost no effect on normal liver cells at low concentrations. Thus, Bel-7402 and Bel-7404 were selected for the subsequent experiments.

We next used flow cytometry to detect two phenotypes related to ferroptosis, cell death, and lipid ROS. Bel-7402 and Bel-7404 were treated with CA for 1 h, and then supplemented with ferroptosis inducer erastin or RSL3. Cell death was detected by SYTOX-Green and the results showed that CA pretreatment combined with ferroptosis inducer has more cell death than with ferroptosis inducer alone (Fig. 1C, D). Cell death was reversed by ferroptosis inhibitor Fer-1. To determine the lipid ROS in liver cancer cells post-treatment, we used C11-BODIPY staining and then perform flow cytometry detection. The results showed that CA alone has no effect on lipid ROS production. Consistent with the cell death results, we found that CA pretreatment for 1 h could produce more lipid ROS, and can be corrected by Fer-1 (Fig. 1E, F). Taken together, we showed that CA can increase the sensitivity of liver cancer cells to ferroptosis.

### Corosolic acid increases the sensitivity of liver cancer cells to ferroptosis by upregulating HERPUD1

**Fig. 2:**
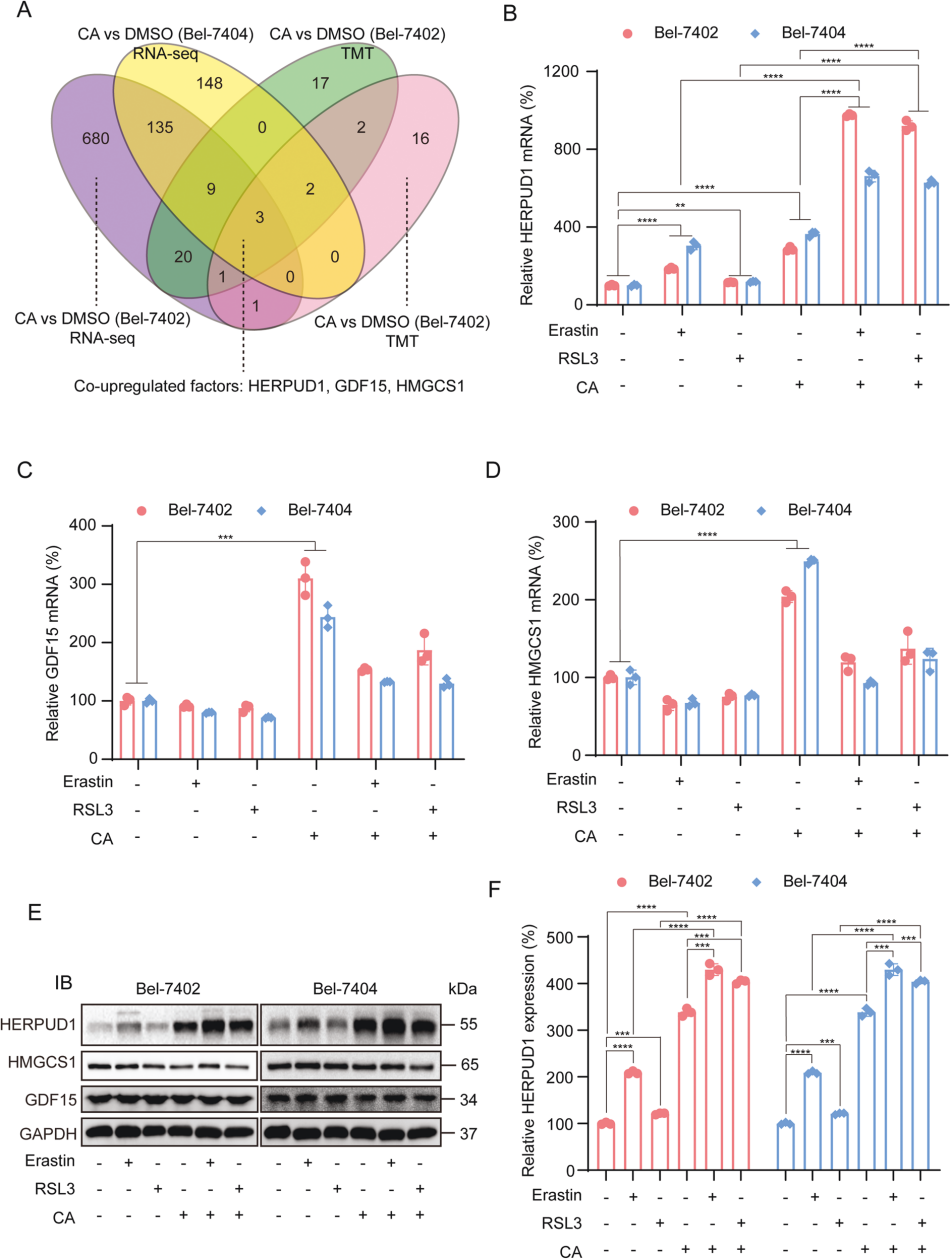
RNA-seq and TMT screened out the gene HERPUD1. **A** Three genes that were co-upregulated were identified by RNA-seq and TMT in Bel-7402 and Bel-7404 cells. **B**–**D** The candidates were further screened by adding CA and ferroptosis inducers. qPCR experiment was used to detect the mRNA of these three candidate genes after adding drugs. **E** HERPUD1, HMGCS1, and GDF15 expression were measured by immunoblotting in liver cancer cell lines (Bel-7402 and Bel-7404), as indicated. The IB data are representative images from three biological replicates. **F** Densitometric analysis of HERPUD1 protein expression in Bel-7402 and Bel-7404 cells normalized to housekeeping protein GAPDH. Data were analyzed using Student’s *t* test and expressed as mean ± SD. ***P < 0.001; ****P < 0.0001.

To identify the key genes that enable CA to increase ferroptosis sensitivity, high-throughput omics for sequencing were utilized. As shown in Fig. 2A, compared with the control group, after CA treatment, we screened out three co-upregulated genes, namely HERPUD1, 3-hydroxy-3-methylglutaryl-CoA synthase 1 (HMGCS1), growth differentiation factor 15 (GDF15) through transcriptome sequencing technology and tandem mass tags (TMT). To determine the most critical gene, we first pretreated with CA for 1 h, before treatment with ferroptosis inducer erastin or RSL3. mRNA expression of the three genes was quantified by qPCR. All three genes were all upregulated after CA treatment, further confirming the high-throughput omics results (Fig. 2B–D). Interestingly, we found that relative HERPUD1 mRNA level was significantly higher when pretreated with CA compared with CA or ferroptosis inducers alone, while GDF15 and HMGCS1 did not show the same tendency. Therefore, we hypothesized that CA sensitizes ferroptosis through HERPUD1. Immunoblotting was used to detect the expression of HERPUD1 after the treatment. The results showed that the HERPUD1 protein level increased when pretreated with CA, compared with the control group, with CA or ferroptosis inducer alone, but HMGCS1 and GDF15 did not show this trend (Fig. 2E). From gene and protein levels, we showed that CA increased the sensitivity of liver cancer cells to ferroptosis by upregulating HERPUD1.

**Fig. 3:**
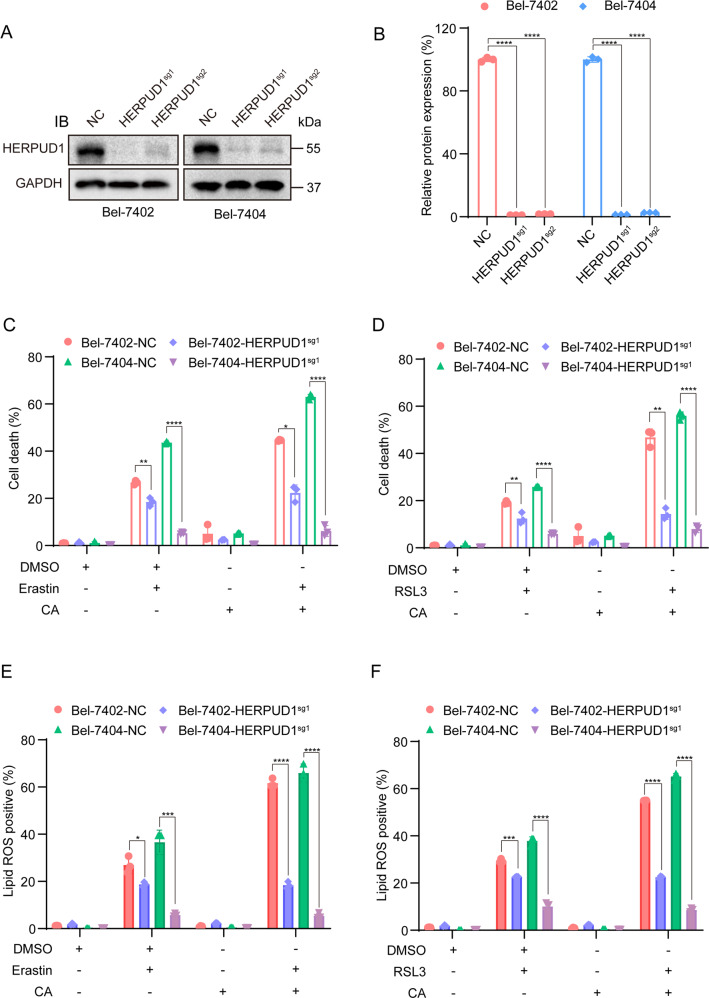
CA increases the sensitivity of HCC cells to ferroptosis by upregulating HERPUD1. **A** Immunoblotting analysis of the expression of HERPUD1 in liver cancer cells after Crispr–Cas9-mediated HERPUD1 depletion. **B** The relative protein level of HERPUD1 is normalized according to the level of GAPDH calculated using ImageJ software. **C**, **D** Bel-7402 and Bel-7404 cells with either wild-type or HERPUD1-sg1 were seeded at an indicated density in six-well plates and cultured for 24 h, then treated with: 10 μM erastin or 5 μM RSL3; 10 μM CA; pretreatment with 10 μM CA for 1 h followed by 10 μM erastin or 5 μM RSL3, in complete medium for 24 h. Cell death was quantified by flow cytometry. **E**, **F** Lipid ROS measurement of HERPUD1-sg1 and the wild-type cells seeded in six-well plates after treated with: 10 μM erastin or 5 μM RSL3; 10 μM CA; pretreatment with 10 μM CA for 1 h followed by 10 μM erastin or 5 μM RSL3, in complete medium for 10 h. Data were analyzed using Student’s *t* test and expressed as mean ± SD. *P < 0.05; **P < 0.01; ***P < 0.001; ****P < 0.0001.

Next, we examined whether HERPUD1 played a vital role in increasing the sensitivity of liver cancer cells to ferroptosis following CA treatment. Firstly, Crispr–Cas9 was used to construct cells that stably knocked out HERPUD1. The knockout efficiency of HERPUD1 was verified in two liver cancer cell lines (Fig. 3A, B). The first knockout (HERPUD1-sg1) was more efficient and was used in further downstream analysis. Flow cytometry was used to observe whether the two phenotypes related to ferroptosis changed after knocking out HERPUD1. We treated WT and HERPUD1-sg1 liver cancer cells: ferroptosis inducer treatment group, CA treatment group, CA and ferroptosis inducers combined treatment group. After 24 h of treatment, cell death was quantified by flow cytometry. Compared with WT liver cancer cells, cell death was reduced in HERPUD1-sg1 cells after treatment (Fig. 3C, D). Similarly, after the same drug treatment for 10 h, the production of lipid ROS was also reduced in HERPUD1-sg1 cells (Fig. 3E, F). Interestingly, the same number of cell death or lipid ROS were produced in HERPUD1-sg1 cells when erastin or RSL3 were added after 1 h of CA pretreatment compared to wild-type liver cancer cells. Liver cancer cells that knockout HERPUD1 produced less lipid reactive oxygen species and cell death after treatment with the CA or ferroptosis inducers. In summary, we can infer from the above results that CA increases the sensitivity of liver cancer cells to ferroptosis by upregulating HERPUD1.

### Corosolic acid inhibits glutathione synthesis through the gene HERPUD1

**Fig. 4:**
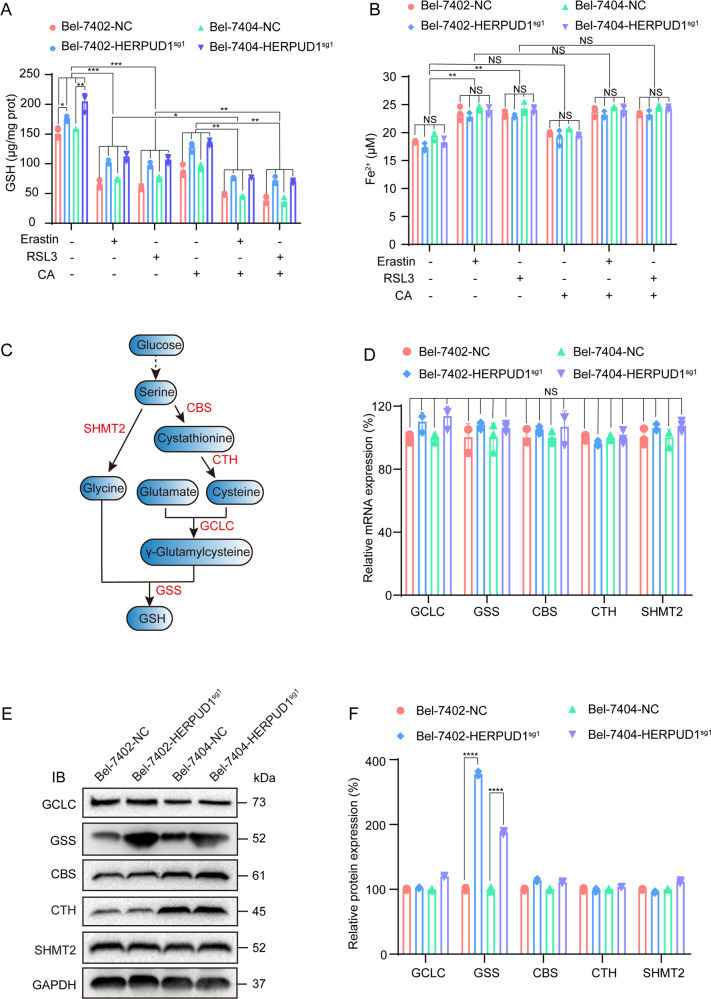
HERPUD1 downregulates GSH by reducing the protein level of GSS. **A**, **B** GSH and Fe^2+^ content in liver cancer cells with or without HERPUD1 after adding erastin, RSL3, CA. **C** The ferroptosis-related metabolic axis from glucose to GSH. **D** mRNA levels of GCLC, GSS, CBS, CTH, and SHMT2 were analyzed by qPCR in Bel-7402 and Bel-7404 cells with or without HERPUD1. **E** Protein levels of GCLC, GSS, CBS, CTH, and SHMT2 were analyzed by immunoblotting in Bel-7402 and Bel-7404 cells with or without HERPUD1. **F** The relative protein level of GCLC, GSS, CBS, CTH, and SHMT2 are normalized according to the level of GAPDH calculated by ImageJ software. The data were analyzed using Student’s *t* test and shown as the mean ± SD from three biological replicates. *P < 0.05; **P < 0.01; ***P < 0.001 indicates statistical significance.

Ferroptosis is caused by the inhibition of the glutathione (GSH) synthesis pathway, the large accumulation of iron, and excessive lipid synthesis [27]. Ferrous ions (Fe^2+^) can initiate liposome peroxidation through Fenton reaction to promote ferroptosis [28]. Therefore, the degree of ferroptosis can be quantified by detecting the content of Fe^2+^. Similarly, ferroptosis can also be detected by quantifying the content of GSH. We confirmed that CA increases the sensitivity of liver cancer cells to ferroptosis through HERPUD1. We next investigated the mechanism of how CA increases the sensitivity of liver cancer cells to ferroptosis through HERPUD1. We tested GSH and Fe^2+^ contents in WT and HERPUD1-sg1 liver cancer cells after different treatments. The content of GSH was decreased after adding ferroptosis inducers erastin or RSL3 to WT liver cancer cells. Moreover, the results showed that GSH decreased more significantly after CA pretreatment (Fig. 4A). In HERPUD1-sg1 liver cancer cells, the content of GSH in the same treatment group was significantly higher than that in WT liver cancer cells (Fig. 4A). The Fe^2+^ content in WT liver cancer cells increased after adding erastin or RSL3, but the Fe^2+^ content did not increase significantly after CA pretreatment compared with adding ferroptosis inducer alone (Fig. 4B). In addition, Fe^2+^ content in the same treatment did not change significantly in WT and HERPUD1-sg1 liver cancer cells (Fig. 4B). Therefore, we preliminarily inferred that CA increases ferroptosis sensitivity through HERPUD1 by reducing the content of intracellular GSH. We then further explored how HERPUD1 affects GSH content. GSH is synthesized from glycine, cysteine, and glutamate [29, 30], and cystine and cysteine can be produced by the metabolic axis of glucose to serine [31, 32]. The GSH synthesis pathway and the pivotal enzymes required in the synthesis process are shown in Fig. 4C. We examined the enzymes that participate in GSH metabolism to investigate which is essential for HERPUD1 to regulate GSH. These enzymes include cystathionine beta-synthase (CBS), cystathionine gamma-lyase (CTH), serine hydroxymethyltransferase 2 (SHMT2), glutamate-cysteine ligase catalytic subunit (GCLC) and glutathione synthetase (GSS) (Fig. 4C). We first designed primers for these enzymes and then used qPCR to detect whether the mRNA expression of these enzymes changed in HERPUD1-sg1 cells compared with WT cells. The results showed that the expression of enzymes did not change at the mRNA level (Fig. 4D). Immunoblotting experiments were used to detect the changes of these enzymes at the protein level. Compared with wild-type cells, the expression of GSS protein increased after the HERPUD1 gene was knocked out, and there were no significant changes in other proteins (Fig. 4E). To sum up, we can infer that CA increases ferroptosis sensitivity through HERPUD1 mainly by reducing the protein level of GSS and reducing the synthesis of GSH.

### CA regulates GSS content by reducing MDM2 ubiquitination through HERPUD1

**Fig. 5:**
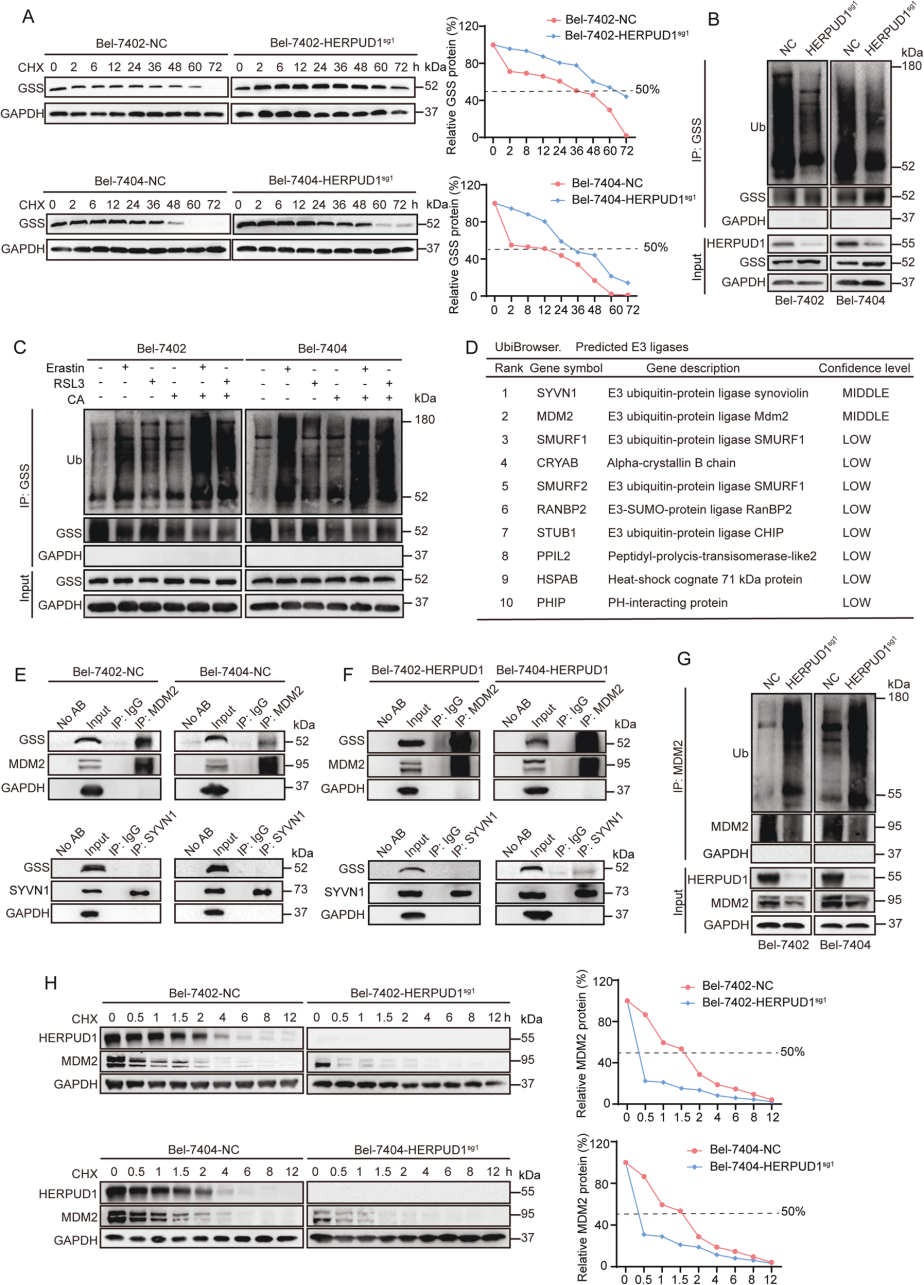
HERPUD1 affects the ubiquitination of GSS by acting on MDM2. **A** Half-life of GSS in Bel-7402 or Bel-7404 cells with or without HERPUD1 knocked out was detected by CHX chase experiments. The relative protein levels of GSS were normalized to those of GAPDH, and the “0 h” point was arbitrarily set to 100%. **B** Total ubiquitination of GSS was detected by co-IP experiments and measured by an anti-total ubiquitin antibody. **C** Bel-7402 and Bel-7404 cells were seeded at the indicated density in 10 cm petri dish and cultured for 24 h, then treated with: 10 μM erastin or 5 μM RSL3; 10 μM CA; pretreatment with 10 μM CA for 1 h followed by 10 μM erastin or 5 μM RSL3, in complete medium for 24 h. The drug-treated protein was then collected, co-IP was performed with anti-GSS antibody according to the previous steps, and measured by anti-total ubiquitin antibody. **D** UbiBrowser online software and the literature was used to identify GSS-related ubiquitinating enzymes. **E** Reciprocal IP experiments of GSS, MDM2 and SYVN1 in wild-type Bel-7402 and Bel-7404 cells. **F** Reciprocal IP experiments for detecting GSS, MDM2 and SYVN1 in Bel-7402 and Bel-7404 cells which HERPUD1 were overexpressed. **G** Endogenous MDM2 was immunoprecipitated by anti-MDM2 antibody and ubiquitination of MDM2 were measured by anti-ubiquitin antibody in Bel-7404 and Bel-7402 cells with or without HERPUD1 knocked out. Representative images of IB are shown in the figure. **H** Half-life of MDM2 was detected by CHX chase experiments in Bel-7402 and Bel-7404 cells with or without HERPUD1 knocked out. The relative protein levels of MDM2 were normalized to those of GAPDH, and the “0 h” point was arbitrarily set to 100%. Data were expressed as mean ± SD. Images of all the immunoblots are representative of three independent experiments.

We have shown that CA inhibits the synthesis of GSH by reducing GSS through HERPUD1. We will further clarify the specific mechanism by which CA affects GSS through HERPUD1. The results of cycloheximide (CHX) chase experiment showed that the half-life of GSS was prolonged in liver cancer cells after HERPUD1 was knocked out (Fig. 5A). Then, we need to explore why the half-life of GSS is prolonged after HERPUD1 is knocked out. HERPUD1 is upregulated in response to endoplasmic reticulum stress [33]. It is reported that HERPUD1 is related to ubiquitination [34, 35], so we hypothesized HERPUD1 reduces the content of GSS by affecting the ubiquitination of GSS. Co-IP experiment was used to verify whether the ubiquitination level of GSS has changed in WT or HERPUD1-sg1 liver cancer cells. We found that after knocking out HERPUD1, the ubiquitination of GSS was reduced compared with the control group (Fig. 5B). We further added ferroptosis inducer and CA to detect how the ubiquitination of GSS changes in liver cancer cells. The ubiquitination of GSS increased after erastin and RSL3 were added (Fig. 5C). When cells were pretreated with CA followed by erastin and RSL3, the ubiquitination of GSS increased more significantly (Fig. 5C). Based on this finding, we considered whether HERPUD1 could increase the ubiquitination of GSS by affecting the ubiquitinating enzyme associated with GSS. GSS-related ubiquitinating enzymes were retrieved using the UbiBrowser software. Two major E3 ubiquitination ligases SYVN1 and MDM2 were selected (Fig. 5D). Co-IP experiments were used to verify whether GSS interacts with these two ubiquitinating enzymes. The results demonstrated that GSS interacts with MDM2, but not with SYVN1 (Fig. 5E). We also tested whether the binding between GSS and the two ubiquitinases changed following HERPUD1 overexpression. The results demonstrated that after HERPUD1 is overexpressed, the binding between GSS and MDM2 becomes stronger, but the relationship between GSS and SYVN1 does not change (Fig. 5F). According to reports, murine double minute 2 (MDM2) is an oncoprotein [36–38] and can induce the ubiquitination of multiple target proteins [39]. Compared with normal liver cancer cells, the ubiquitination of MDM2 was increased in HERPUD1-sg1 liver cancer cells by co-IP experiments (Fig. 5G), and the half-life of MDM2 was prolonged in liver cancer cells knocked out HERPUD1 by CHX chase experiments (Fig. 5H). These results demonstrate that CA increases the sensitivity of liver cancer cells to ferroptosis by upregulating HERPUD1, while HERPUD1 reduces the ubiquitination of MDM2 to reduce the content of GSS, thereby inhibiting GSH synthesis and promoting ferroptosis.

### In vivo experiments showed that CA increased ferroptosis sensitivity by upregulating HERPUD1

**Fig. 6:**
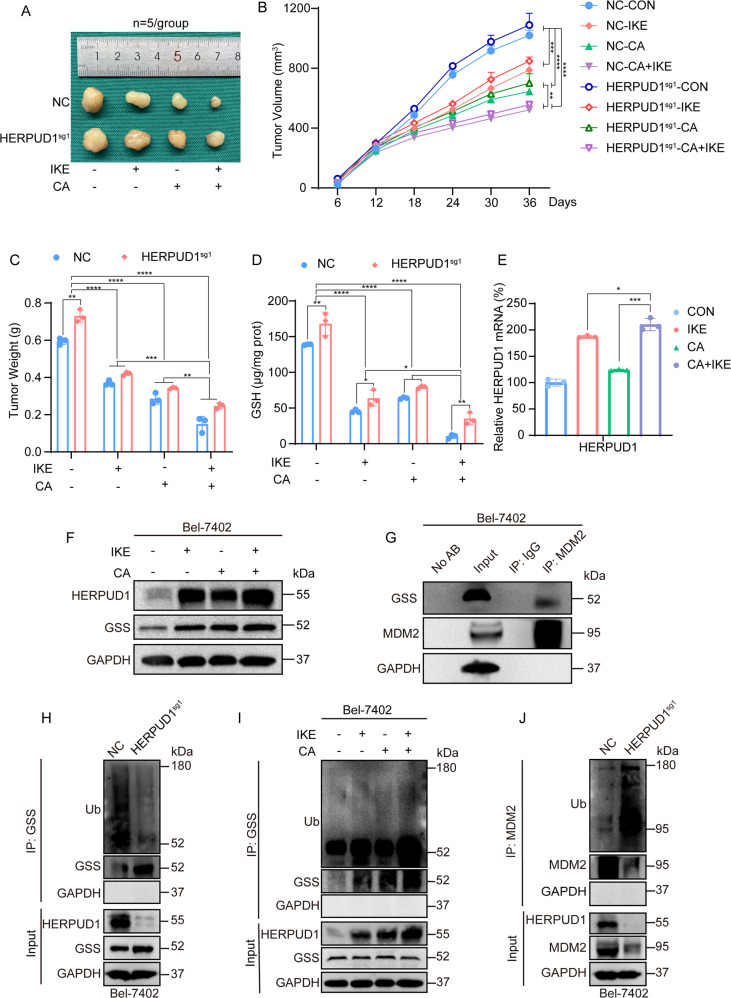
In vivo experiments demonstrate that CA increases ferroptosis sensitivity through HERPUD1. **A** Representative tumor pictures after CA (10 mg/kg), IKE (50 mg/kg), CA and IKE combined treatment (*n* = 5/group). **B** Tumor growth curve monitored for 36 days, tumor volume (mm^3^) = 0.5 × length × width^2^. **C** Tumors weighed after 36 days. **D** GSH content in tumors was detected after drug treatment using a GSH kit. **E** mRNA levels of HERPUD1 in tumors were analyzed by qPCR assay after drug treatment. **F** Protein levels of HERPUD1 in tumors were analyzed by immunoblotting. Representative images of IB were shown in the figure. **G** The interaction of GSS and MDM2 in tumors were detected by co-IP experiments. **H** Total ubiquitination of GSS, as co-IP by anti-GSS antibody for the indicated treatment in Bel-7402 cells with or without HERPUD1 knocked out, and measured by anti-total ubiquitin antibody. **I** Total ubiquitination of GSS, as co-IP by anti-GSS antibody for the indicated treatment in Bel-7402 cells with or without drug treatment, and measured by anti-total ubiquitin antibody. **J** Total ubiquitination of MDM2, as co-IP by anti-MDM2 antibody for the indicated treatment in Bel-7402 cells with or without HERPUD1 knocked out, and measured by anti-total ubiquitin antibody. Images of all the immunoblots are representative of three independent experiments. Data were analyzed using Student’s *t* test and expressed as mean ± SD from three biological replicates. *P < 0.05; **P < 0.01; ***P < 0.001 indicates statistical significance.

Previously, we demonstrated through in vitro cell experiments that CA acts on MDM2 via HERPUD1 to reduce GSS and then inhibit GSH synthesis, thereby increasing ferroptosis. Next, we verified whether CA has the same effect in vivo using xenograft model experiments. In vivo experiments showed that CA and ferroptosis inducer IKE could inhibit the growth of xenografts, and the inhibition effect was more significant when combined (Fig. 6A–C). The results shown that after the same drug treatment, the size of HERPUD1-sg1 xenografts is larger than that of wild-type, indicating that ferroptosis is reduced after knockout of HERPUD1, and the inhibitory effect of drugs on xenografts is weakened (Fig. 6A). We randomly removed tissues from each treatment group for grinding, and detected the content of GSH, we found that the content of GSH decreased after adding CA and the ferroptosis inducer IKE, and the reduction of GSH content was more significant when the two drugs were combined (Fig. 6D). However, after the same drug treatment, the GSH content of each group knocking out HERPUD1 was generally higher than that of the wild-type (Fig. 6D), which was consistent with the in vitro results. The results show we detected the expression of HERPUD1 at the mRNA and protein levels (Fig. 6E, F), which were consistent with the previous in vitro results. The interaction between GSS and MDM2 in tissues detected by co-IP experiments (Fig. 6G), which was consistent with the experimental results in vitro. The ubiquitination of GSS was reduced, and the ubiquitination of MDM2 was increased after knockout HERPUD1 (Fig. 6H, J). The ubiquitination of GSS increased after the addition of CA and the ferroptosis inducer IKE, and the increase in ubiquitination was more pronounced after these two drugs were combined. Overall, the results of the in vivo experiments are consistent with our previous in vitro experiments. Therefore, we confirmed from both in vivo and in vitro experiments that CA acts on MDM2 through HERPUD1 to reduce GSS, and then inhibit the synthesis of GSH to increase ferroptosis.

## Discussion

Liver cancer remains a major global health challenge with rising incidence worldwide and high mortality, according to estimates by the World Health Organization (WHO) [2]. Standard chemotherapy is ineffective for most patients with liver cancer, finding more effective treatment methods and drugs is very urgent. Sorafenib, an oncogenic kinase inhibitor, is an FDA-approved drug for the treatment of advanced HCC and has been shown to prolong overall survival in unresectable HCC [40]. The artemisinin derivative dihydroartemisinin can sensitize cancer cells to ferroptosis by increasing free iron levels [41]. CA has received extensive attention due to its various pharmacological activities such as anti-inflammatory, antitumor and anti-diabetic, among which the antitumor effect has become a research hotspot. A previous study [42] showed that CA has anti-HCC effects both in vitro and in vivo for the treatment of HCC cells. In this study, we found that the combination of CA with ferroptosis inducers can increase the sensitivity of liver cancer cells to ferroptosis (Fig. 1).

The key gene HERPUD1 that CA increases the ferroptosis sensitivity of liver cancer cells was screened by RNA-seq and TMT (Fig. 2). HERPUD1 is an endoplasmic reticulum (ER) membrane protein that is generally expressed and highly induced during unfolded protein responses (UPR) and cell stress [35]. HERPUD1 plays a role in the regulation of cellular homeostasis and protein degradation through the formation of endoplasmic reticulum-associated degradation (ERAD) complex in the proteasomal pathway [33]. HERPUD1 is a recognized UPR target, a component and regulator of ERAD [43], and E3 ubiquitin ligase HRD1 [44, 45], ubiquitin [45], SEL1L, OS-9 and Derlin-1 [44] interact to degrade a variety of proteins. A previous study showed that Circ_002117 upregulated HERPUD1 through miR-370 binding to increase the apoptosis of gastric cancer cells induced by ER stress [46]. HERPUD1 reduces apoptosis by reducing the content of inositol 1,4,5-trisphosphate receptor (ITPR) [33]. However, the role of HERPUD1 in liver cancer and ferroptosis is less clear. In this study, we found that CA increased the sensitivity of liver cancer cells to ferroptosis by upregulating HERPUD1 (Figs. 2 and 3).

Ferroptosis is a regulated form of cell death that is iron-dependent. Recent research has shown that ferroptosis is implicated in numerous human diseases and pathological processes, such as tumors [47], ischemia-reperfusion injury [48], renal damage [49], cardiomyopathy [50], blood diseases [51], and iron metabolism-related diseases [52]. GSH is an important antioxidant, and GSH-related antioxidant systems can provide energy for cell growth and migration. In liver cancer cells, GSH-related antioxidant systems can inhibit ferroptosis [53–55], because GSH can reduce lipid peroxides to the corresponding alcohols and protect cells from lipid peroxide damage [53, 56]. It is also understandable that GSH depletion leads to ferroptosis [56–58]. According to previously published reports, both glycine and cysteine, the raw materials for the synthesis of GSH, can be produced through the metabolic process of converting glucose to serine (Fig. 4C) [31, 32]. Therefore, the maintenance of anti-ferroptotic activity in cancer cells is closely related to the enzyme that converts serine to GSH [59]. For GSS, one study reported that inhibition of GSS by the compound solanine, isolated from *Solanum melongena*, increased lipid ROS levels in HepG2 cells [60]. In our study, we found that CA reduced the protein level of GSS by upregulating HERPUD1 (Fig. 4E) and inhibited the synthesis of GSH, thereby promoting the occurrence of ferroptosis.

**Fig. 7:**
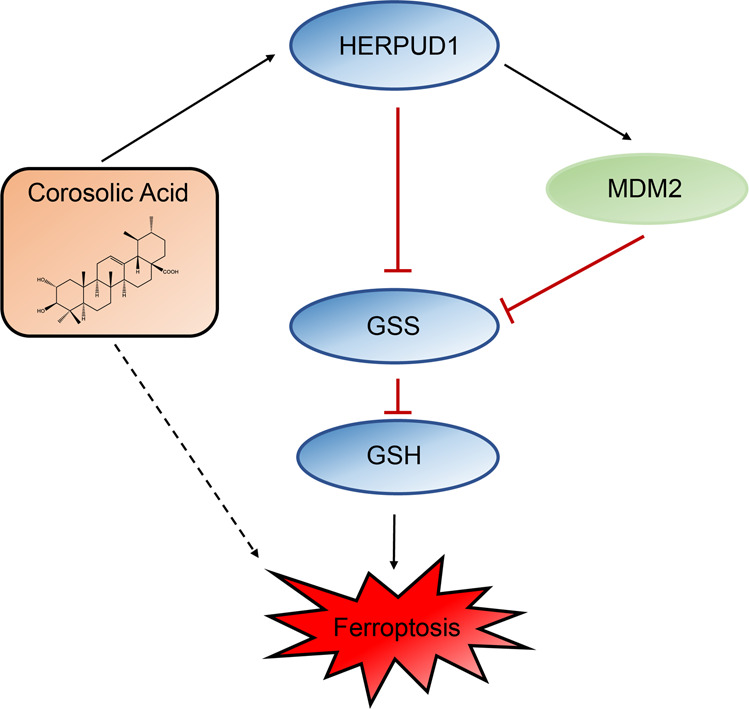
Mechanism diagram of CA increasing ferroptosis sensitivity of liver cancer cells. Briefly, CA sensitizes ferroptosis by upregulating HERPUD1 in liver cancer cells. It was further analyzed that CA acts on MDM2 through HERPUD1, increases the ubiquitination of GSS, and then reduces the content of GSS to reduce the synthesis of GSH, thereby increasing the sensitivity of liver cancer cells to ferroptosis.

We found that HERPUD1 is related to the ubiquitination of GSS (Fig. 5B, C), and further found the related key ubiquitination enzyme MDM2 of GSS by UbiBrowser software (Fig. 5D–F). Murine double minute 2 (MDM2), or HDM2 in humans, is an oncoprotein [36–38]. The MDM2 gene has been shown to be aberrantly upregulated in human tumor cell lines through gene amplification, increased transcription, and enhanced translation [61]. It is known that MDM2 can induce the ubiquitination of multiple target proteins. MDM2 is in cancer cell types, including lung, breast, colorectal adenocarcinoma, and osteosarcoma. MDM2 is elevated in a multiple of cancer cell types, including colorectal adenocarcinoma, breast cancer, lung cancer, and osteosarcoma [36, 62]. Clinically, aberrant upregulation of MDM2 is associated with drug resistance, metastasis, and poor prognosis in liver cancer [63]. We found that HERPUD1 reduces MDM2 ubiquitination in liver cancer cells (Fig. 5G), while MDM2 induces the ubiquitination of GSS, thereby reducing the content of GSS, and finally inhibiting the synthesis of GSH to increase ferroptosis. In our study, we confirmed by in vivo and in vitro experiments that HERPUD1 reduces GSS by acting on MDM2, thereby inhibiting the synthesis of GSH, and ultimately increasing the sensitivity of HCC cells to ferroptosis (Fig. 7).

## Conclusion

In conclusion, our data are the first to show that CA can increase the sensitivity of liver cancer cells to ferroptosis by upregulating HERPUD1. We further explored the mechanism of ferroptosis and found that CA acts on GSS-related ubiquitinating enzyme MDM2 by inducing HERPUD1 expression to reduce GSS and inhibit GSH synthesis, thereby promoting ferroptosis.

## Materials and methods

### Cell culture, vectors, and drug

Liver cancer cell lines Bel-7402, SMMC-7721, Bel-7404, HepG2, and SK-Hep1, and the hepatocyte cell line HL-7702, which were obtained from our previous study [64]. These cell lines were authenticated, and no mycoplasma contamination was detected. Cells were cultured in Dulbecco’s modified Eagle’s medium (DMEM, HyClone, Logan, UT, USA) supplemented with 1% penicillin–streptomycin and 10% fetal bovine serum (FBS, Gibco, Carlsbad, CA, USA). The cells were maintained in a 37 °C incubator containing 5% carbon dioxide. Cells were treated with CA (SC8500, Solarbio, Beijing, China), erastin (E7781, Sigma, St Louis, MO, USA), RAS-selective lethal 3 (RSL3, S8155, Selleck), ferrostatin-1 (Fer-1, SML0583, Sigma), and Cycloheximide (CHX, HY-12320, MCE, Shanghai, China).

### Immunoblotting (IB)

Conventional protocols were used for IB with the details available elsewhere. The following primary antibodies were used for IB: anti-HERPUD1 (1:1000, ab150424, Abcam, Cambridge, UK), anti-GCLC (1:1000, ab190685, Abcam), anti-CBS (1:1000, ab140600, Abcam), anti-CTH (1:1000, ab189916, Abcam), anti-SHMT2 (1:1000, #33443, Cell Signaling Technology (CST), Boston, MA, USA), anti-GSS (1:1000, #ab124811, Abcam) or GSS (H-7) (1:500, sc-166882, Santa Cruz, Heidelberg, Germany), anti-MDM2 (1:1000, ab259265, Abcam), anti-SYVN1 (1:1000, #14773, CST), anti-GAPDH (1:1000, #5174, CST). The membranes were incubated with anti-rabbit IgG, HRP-linked antibody (1:2000, #7071, CST) or anti-mouse IgG, HRP-linked antibody (1:2000, #7076, CST) at room temperature for one hour, and visualized using Clarity Western ECL substrate. The relative expression levels of proteins were determined by densitometry using ImageJ software and were normalized to housekeeping protein.

### Co-immunoprecipitation (co-IP)

Cell lysates were incubated with the corresponding antibodies overnight at 4 °C, followed by incubation with protein A/G beads. Subsequently, the beads were collected after washing with 1 × PBS and double-distilled water, and an appropriate amount of 1 × loading buffer was added for IB. The antibodies used for co-IP were anti-GSS (1:200, #ab124811, Abcam) or GSS (H-7) (1:50, sc-166882, Santa Cruz), anti-MDM2 (1:200, ab259265, Abcam), anti-SYVN1 (1:200, #14773, CST) and anti-ubiquitin (1:200, #3936, CST).

### Quantitative real-time PCR (qRT-PCR)

According to the manufacturer’s instructions, TRIzol reagent (Invitrogen, Carlsbad, CA, USA) was used to extract total RNA. RNA was reverse transcribed into cDNA using a kit from Vazyme (R323-01, Nanjing, China). All qPCR reactions were performed using 2× SYBR Green qPCR Master Mix. The qPCR data is analyzed using the ΔΔCt method: ΔCt = Ct (target gene) − Ct (GAPDH), ΔΔCt = ΔCt (test group) − ΔCt (control group). The relative expression level of the target gene in cells was calculated as follows: relative mRNA level = 2^−ΔΔCt^. The primers are listed in Supplementary Table 1.

### Cell viability

Cell Counting Kit-8 (CCK-8, Beyotime, Shanghai, China) was used to detect cell viability. For CCK-8-based cell viability assays, Bel-7402 and Bel-7404 cells (8000 cells per well) were seeded in 96-well plates. The medium was changed post-treatment to include CCK-8, incubated 30 min at 37 °C, and absorbance was measured at 450 nm.

### Flow cytometry analysis

Different treatments were performed on Bel-7402 and Bel-7404 cells seeded on a 6-well plate at an appropriate cell density. The cells were collected, centrifuged, and washed twice with cold PBS. Cells were resuspended in 200 μL PBS containing BODIPY 581/591 C11 (D3861, Thermo Fisher, Waltham, MA, USA) and incubated at 37 °C in the dark for 10 min. Flow cytometry was used to detect lipid ROS. Cell death was analyzed by staining with SYTOX-Green (KGA261, KEYGEN, Nanjing, China) followed by flow cytometry. Data were analyzed using FlowJo software.

### GSH determination

Micro Reduced Glutathione (GSH) Assay Kit (BC1175, Solarbio) was used to determine the content of GSH, and a microplate reader was used to measure the absorbance at 412 nm according to the manufacturer’s instructions. GSH content were normalized to protein concentration and expressed as μM/mg protein.

### Iron assay

Ferrous iron in wild-type (WT) and HERPUD1 knockout (HERPUD1-sg1) cells was measured using iron assay kit (ab83366, Abcam, Cambridge, MA) according to the manufacturer’s instructions.

### RNA Sequencing (RNA-seq) and tandem mass tags (TMT) analysis

The transcriptome sequencing and analysis were conducted by OE Biotech Co., Ltd. (Shanghai, China). TMT analysis was performed and analyzed by Luming Biotechnology (Shanghai, China).

### Xenograft mouse model

A total of 24 male athymic nude mice (4–6 weeks old) (JieSiJie, Shanghai, China) were randomly divided into four groups. 1 × 10^7^ WT or HERPUD1-sg1 Bel-7402 cells were injected subcutaneously into the back of the mice. At day 14, Imidazole ketone erastin (IKE, 50 mg/kg, S8877, Selleck, Shanghai, China), CA (10 mg/kg), or CA (10 mg/kg) combined with IKE (50 mg/kg) were injected. Tumors were measured every other day. Tumor volume was calculated as follows: 0.5 × length × width^2^. All animal experiments were approved by the Ethics Committee of Shanghai Hospital of Traditional Chinese Medicine and performed according to the NIH Guide for the Care and Use of Laboratory Animals.

### Statistical analysis

All data and error bars are presented as the mean ± SD from at least three independent experiments. All the data were normally distributed. Tests to examine the differences between groups included Student’s *t* test and one-way ANOVA. *P* < 0.05 was regarded as statistically significant. The variance was similar between the groups and was statistically compared.

## Supplementary information

Table S1

Original Data File

## Data Availability

RNA-seq data have been deposited in GEO (accession number: GSE201455), RNA-seq date can be viewed at https://www.ncbi.nlm.nih.gov/geo/query/acc.cgi?acc=GSE201455, and the password is ubevqgeyxtslnsd. TMT data have been deposited in ProteomeXchange Consortium (accession number: PXD033462), TMT data can be viewed at https://www.iprox.cn/page/PSV023.html;?url=16510400506514YTD, and the password is JKhP.
