# Supplementary material for: Corosolic acid sensitizes ferroptosis by upregulating HERPUD1 in liver cancer cells
Source: Cell Death Discov. 2022 Aug 29;8:376. doi: 10.1038/s41420-022-01169-0 (PMC9424261; doi:10.1038/s41420-022-01169-0)
Supplement: Supplementary file 1 — Table S1 [file 41420_2022_1169_MOESM1_ESM.docx]

**Table S1. Sequence of primers and sgRNA were listed.**

**Primers used for qPCR**

| Name | 5'-3' |
| --- | --- |
| HERPUD1-F | ATGGAGTCCGAGACCGAAC |
| HERPUD1-R | TTGGTGATCCAACAACAGCTT |
| HMGCS1-F | GATGTGGGAATTGTTGCCCTT |
| HMGCS1-R | ATTGTCTCTGTTCCAACTTCCAG |
| GDF15-F | ACCTGCACCTGCGTATCTCT |
| GDF15-R | CGGACGAAGATTCTGCCAG |
| GSS-F | TACGGCTCACCCAATGCTC |
| GSS-R | CTATGGCACGCTGGTCAAATA |
| GCLC-F | GGCACAAGGACGTTCTCAAGT |
| GCLC-R | CAGACAGGACCAACCGGAC |
| CTH-F | GGCCTGGTGTCTGTTAATTGT |
| CTH-R | GCCATTCCGTTTTTGAAATGCT |
| CBS-F | GGCCAAGTGTGAGTTCTTCAA |
| CBS-R | GGCTCGATAATCGTGTCCCC |
| SHMT2-F | GCCACGGCTCATCATAGCTG |
| SHMT2-R | AGCAGGTGTGCTTTGACTTCA |
| GAPDH-F | ATCATCCCTGCCTCTACTGG |
| GAPDH-R | GTCAGGTCCACCACTGACAC |

**sgRNA sequence**

| Name | 5'-3' |
| --- | --- |
| HERPUD1-KO1-F | CACCGGAATCCACAGAGGAGCCTGC |
| HERPUD1-KO1-R | AAACGCAGGCTCCTCTGTGGATTCC |
| HERPUD1-KO2-F | CACCGGAGCAGATTCCTCATGGTCA |
| HERPUD1-KO2-R | AAACTGACCATGAGGAATCTGCTCC |
| MDM2-KO1-F | CACCGGTACATCTGTGAGTGAGAAC |
| MDM2-KO1-R | AAACGTTCTCACTCACAGATGTACC |
| MDM2-KO2-F | CACCGGAGAACAGGTGTCACCTTGA |
| MDM2-KO2-R | AAACTCAAGGTGACACCTGTTCTCC |
