# Supplementary figures and images for: Corosolic acid sensitizes ferroptosis by upregulating HERPUD1 in liver cancer cells
Source: Cell Death Discov. 2022 Aug 29;8:376. doi: 10.1038/s41420-022-01169-0 (PMC9424261; doi:10.1038/s41420-022-01169-0)

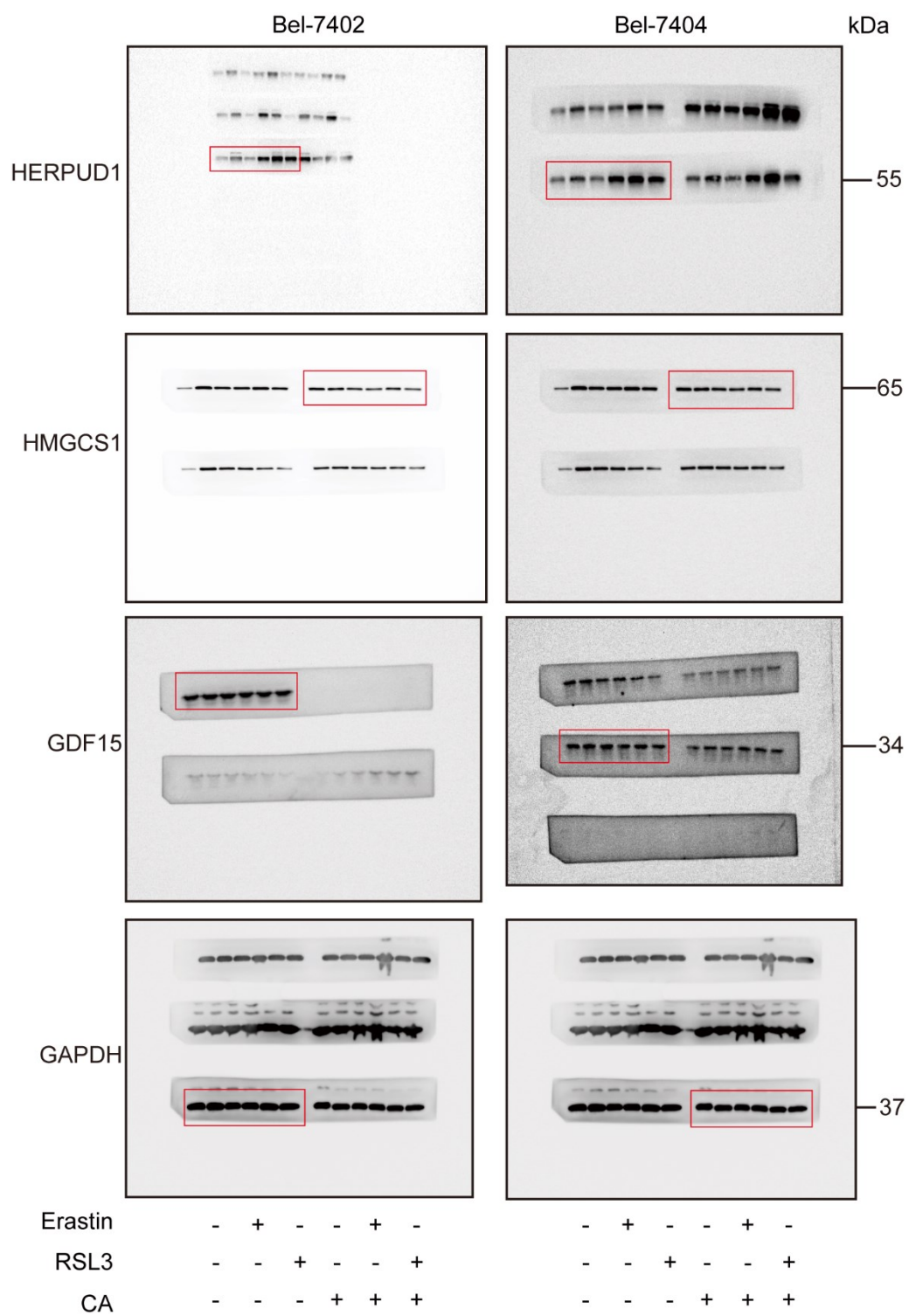

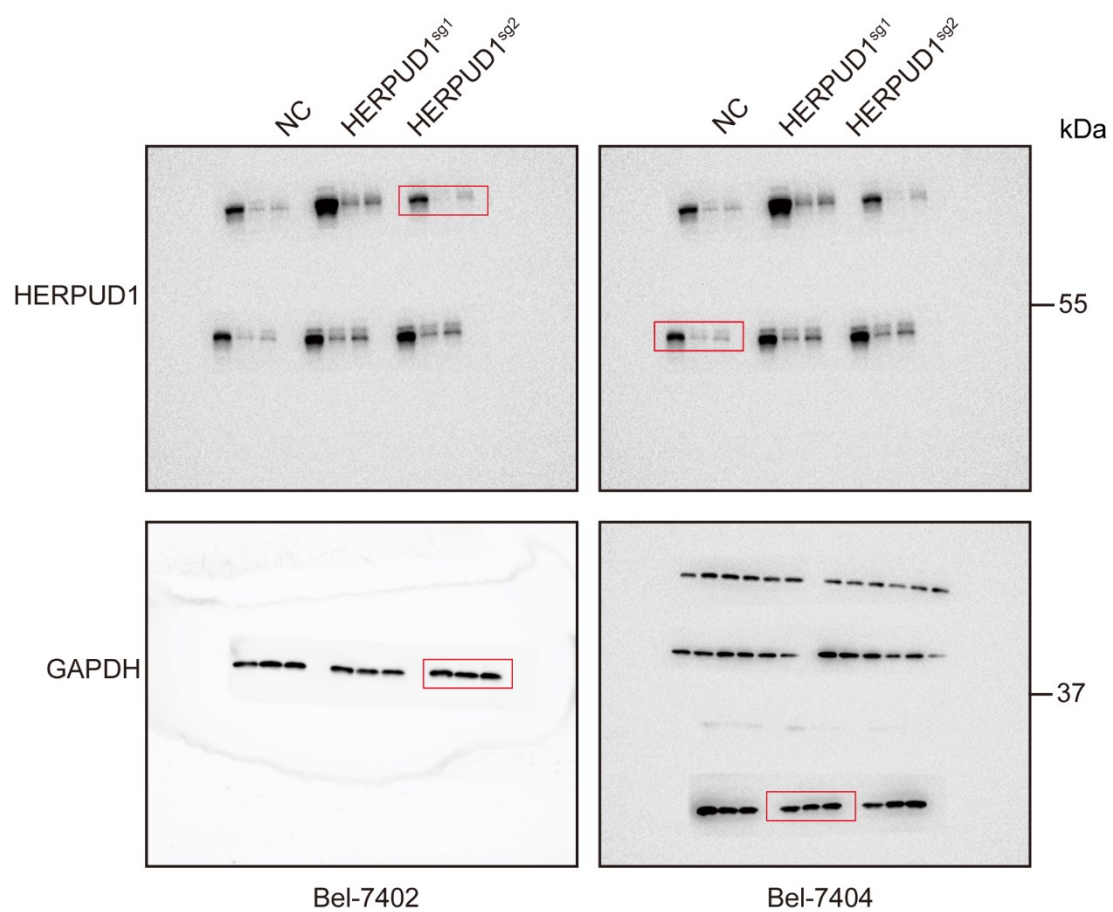

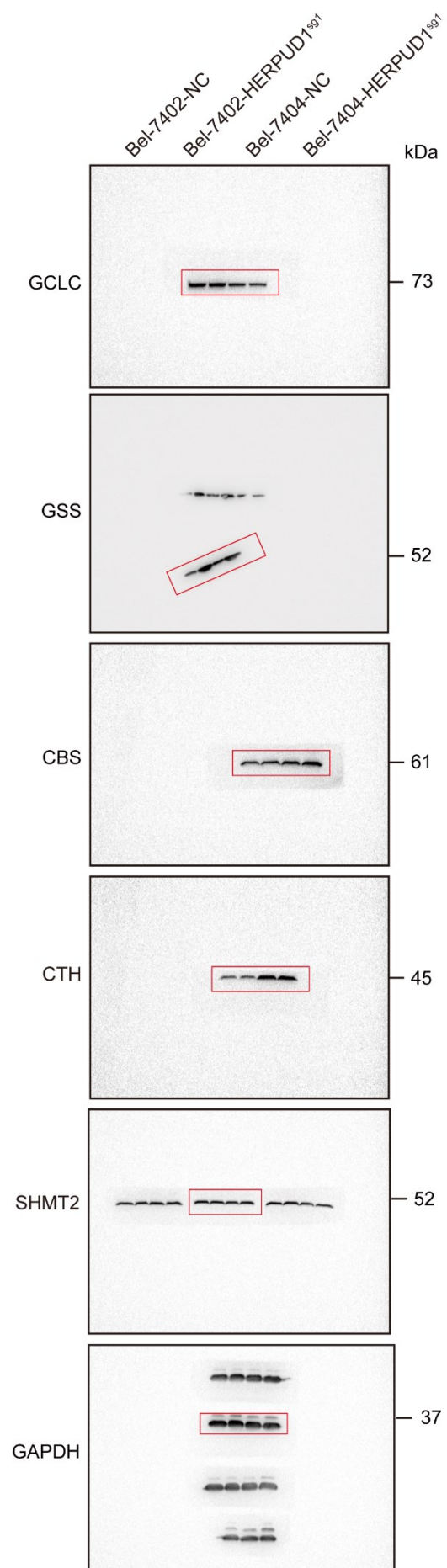

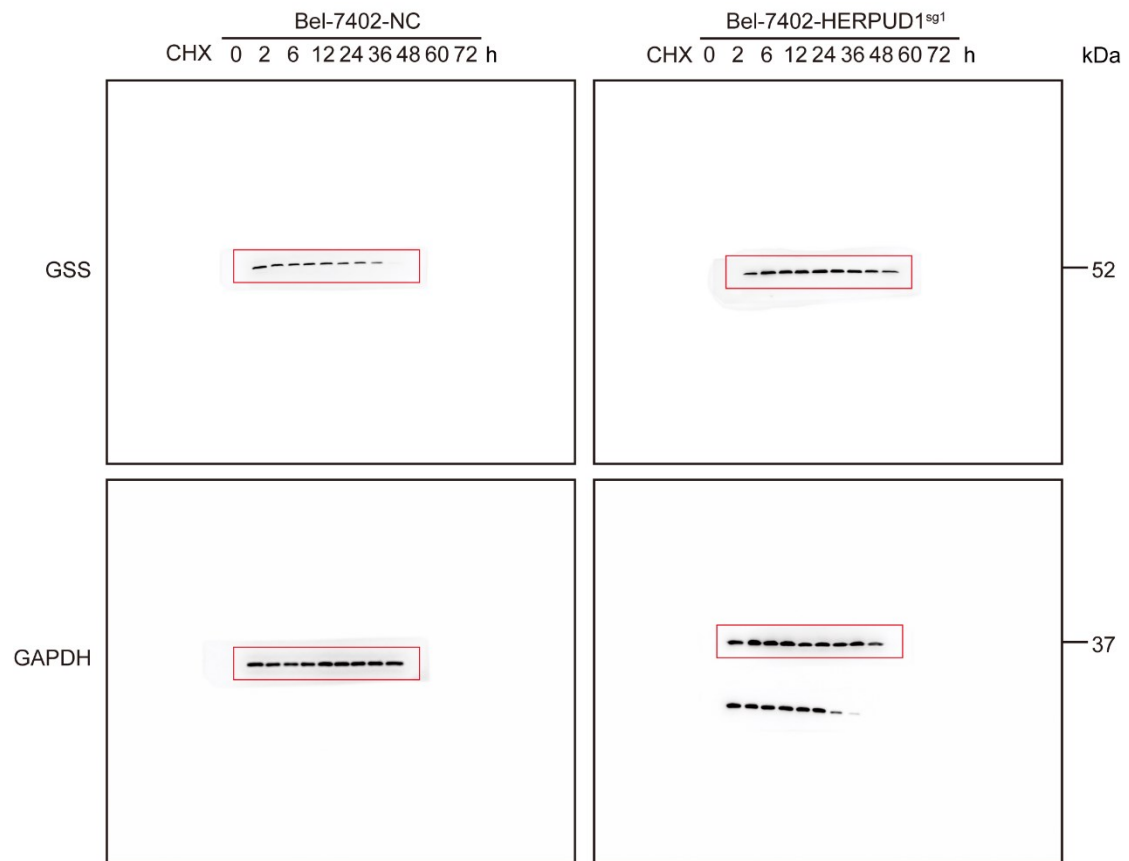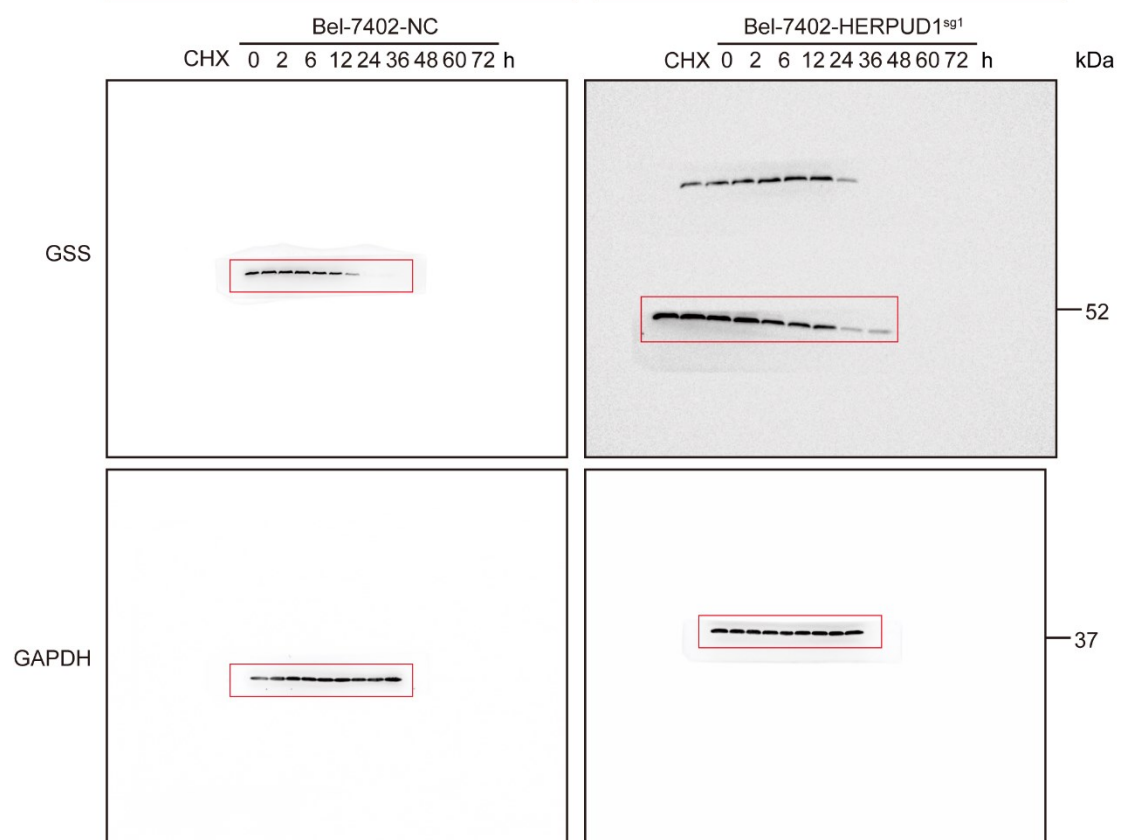

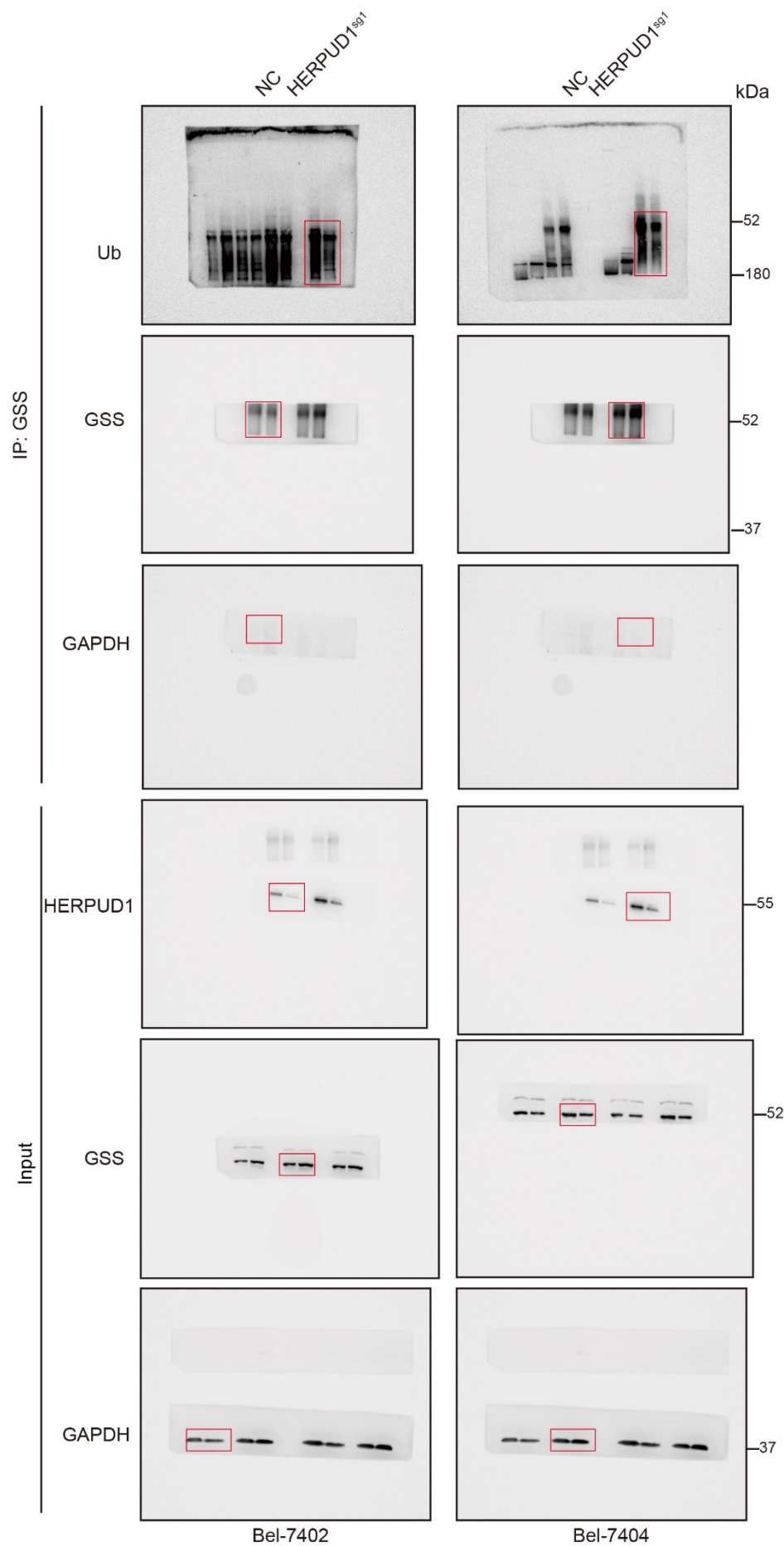

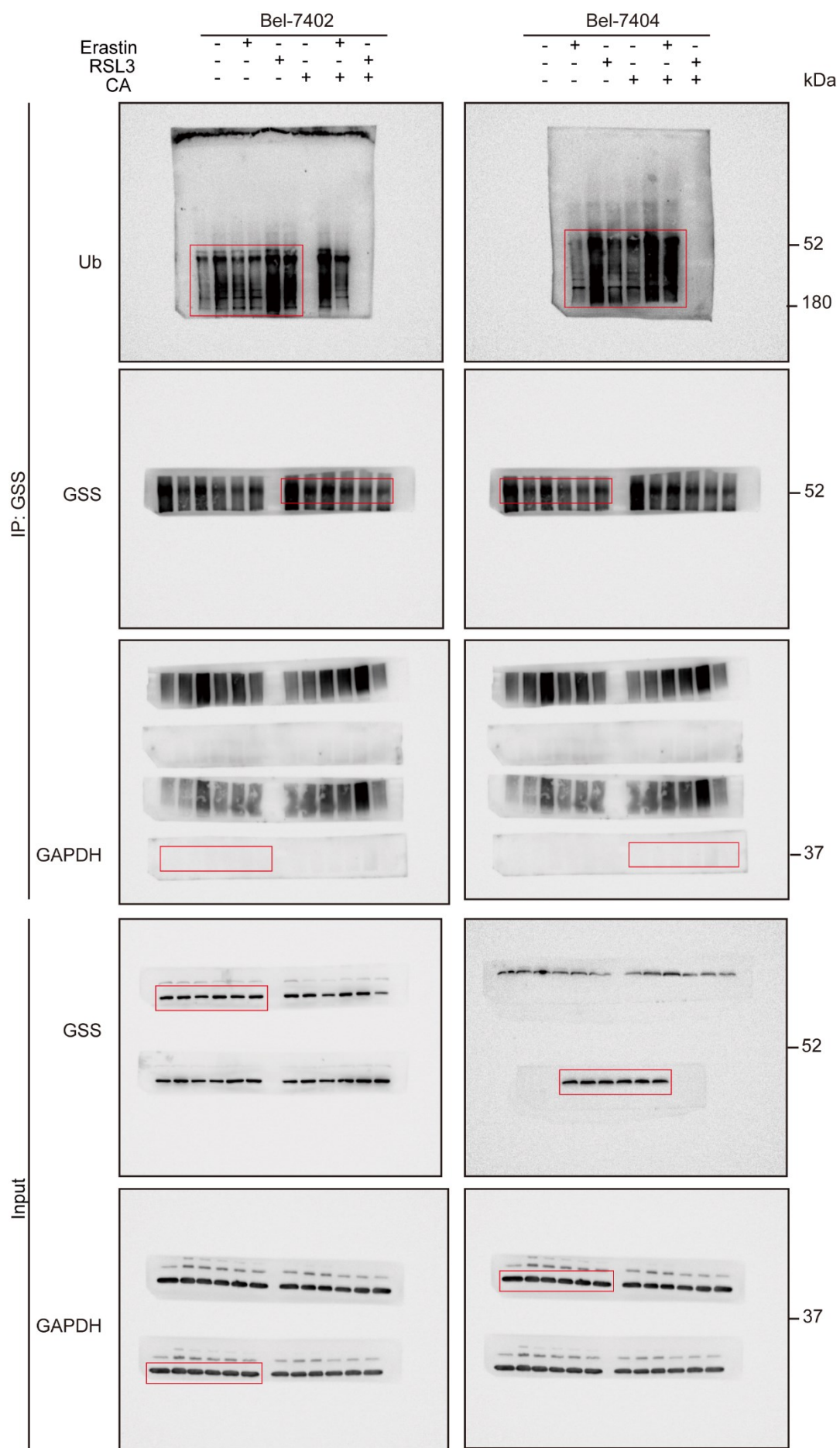

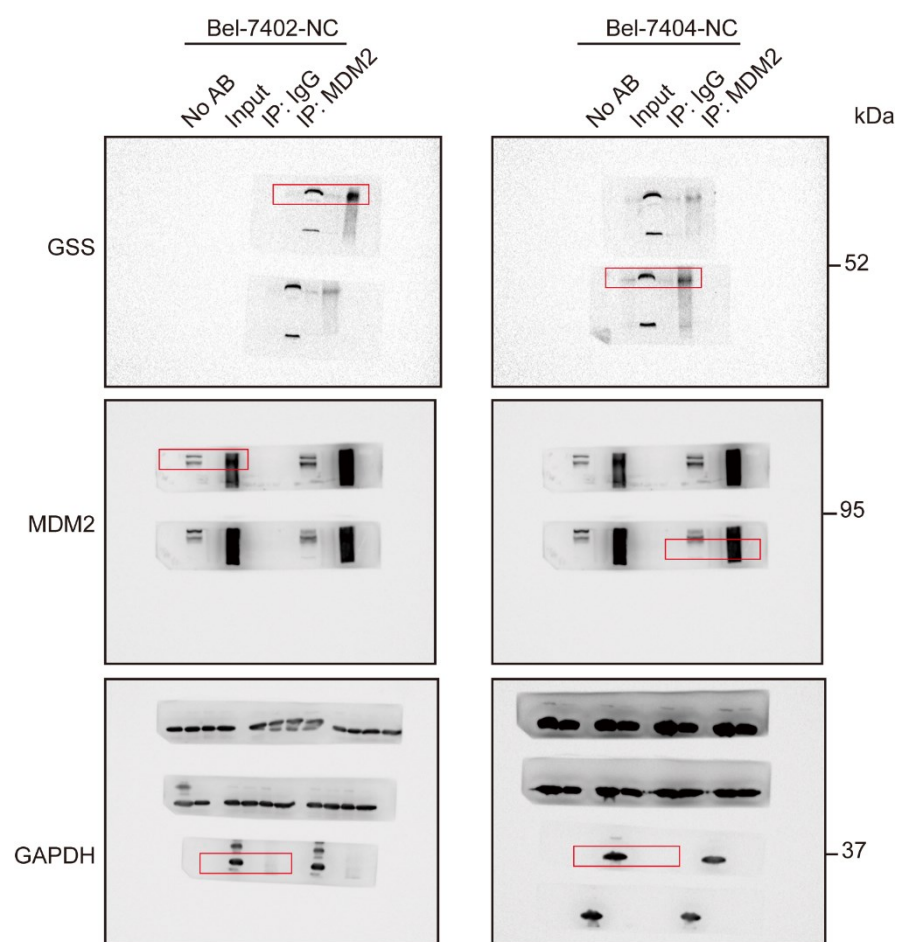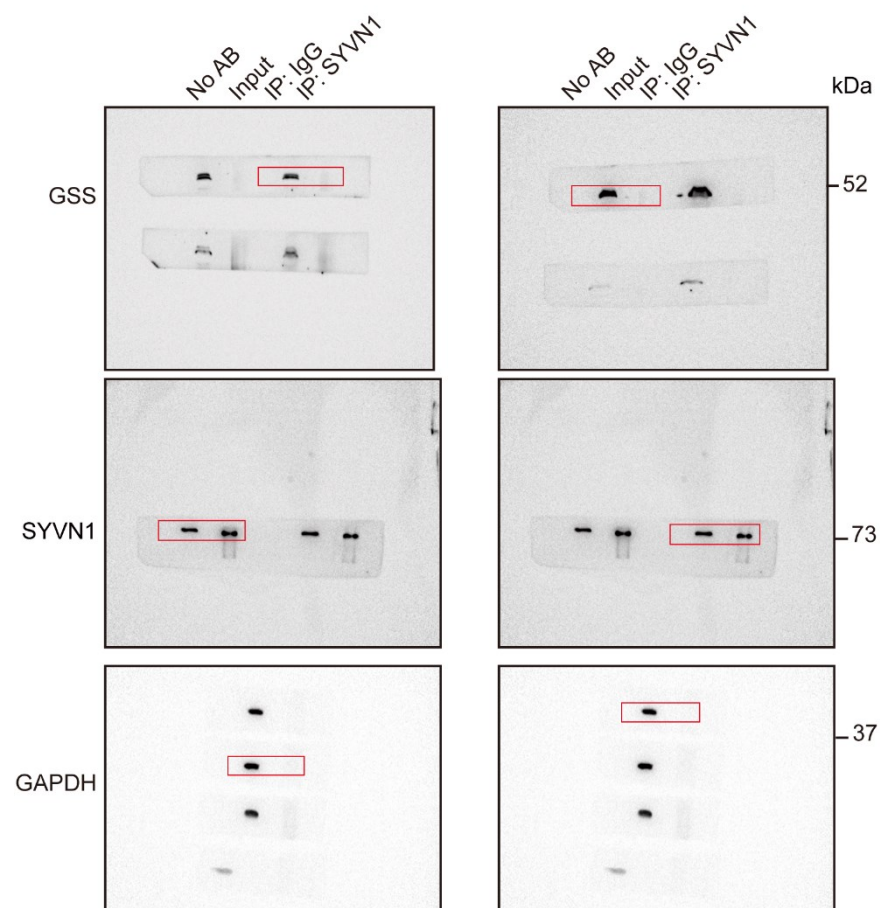

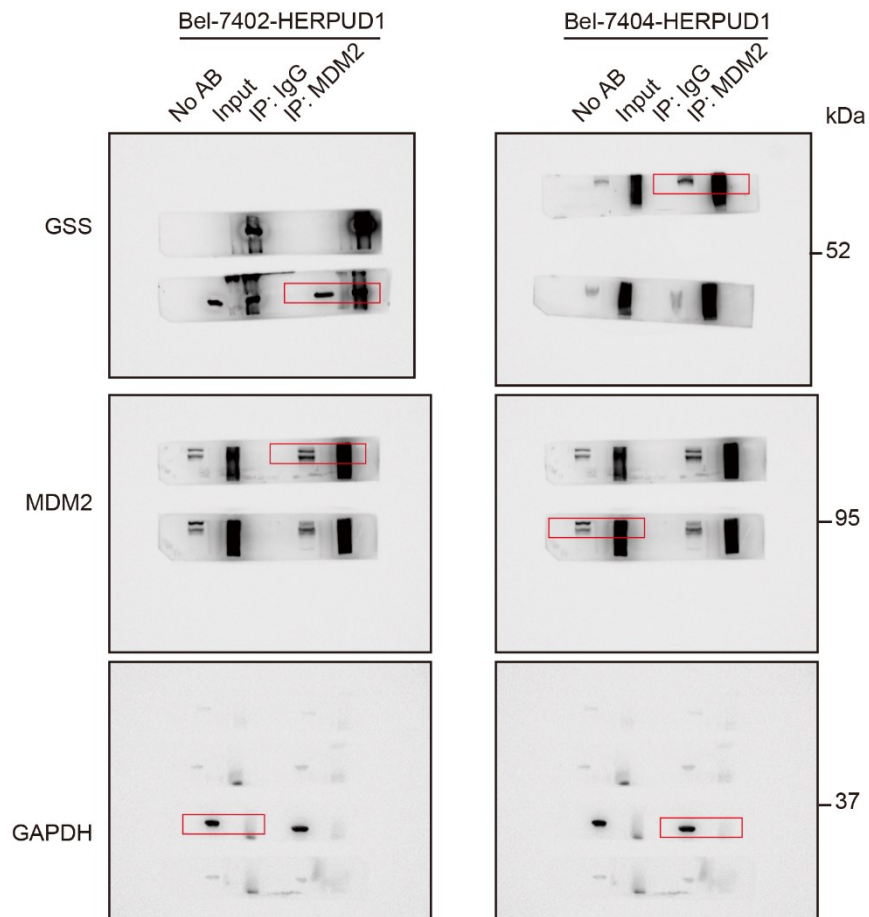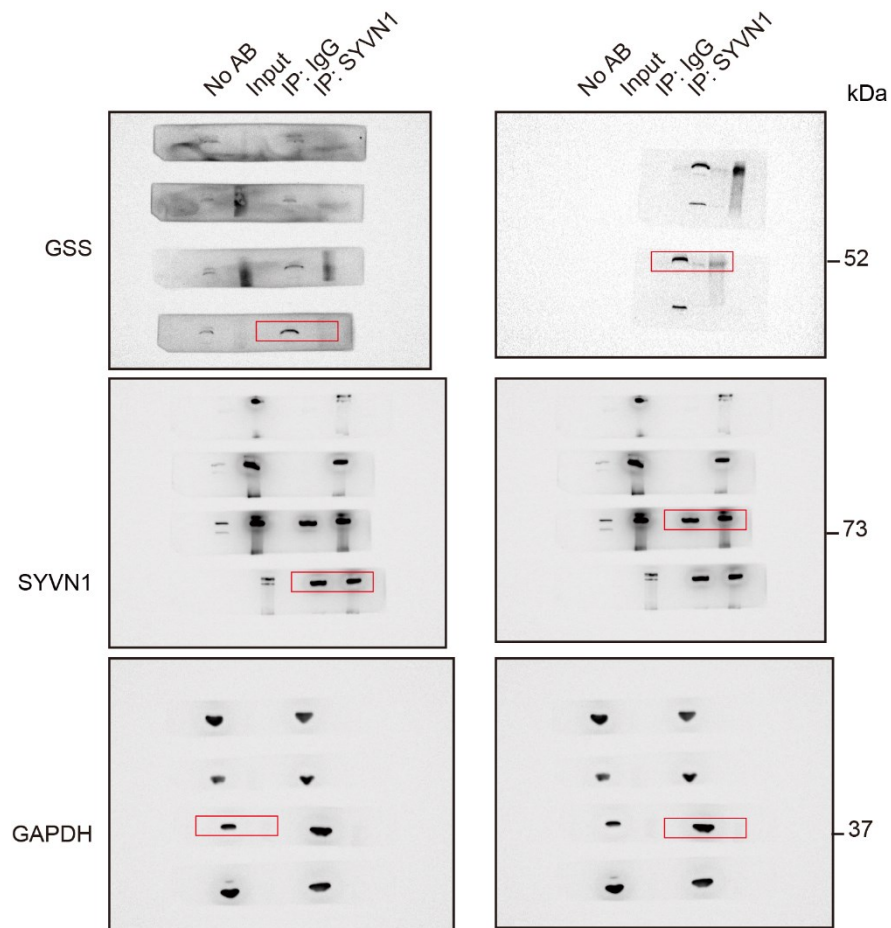

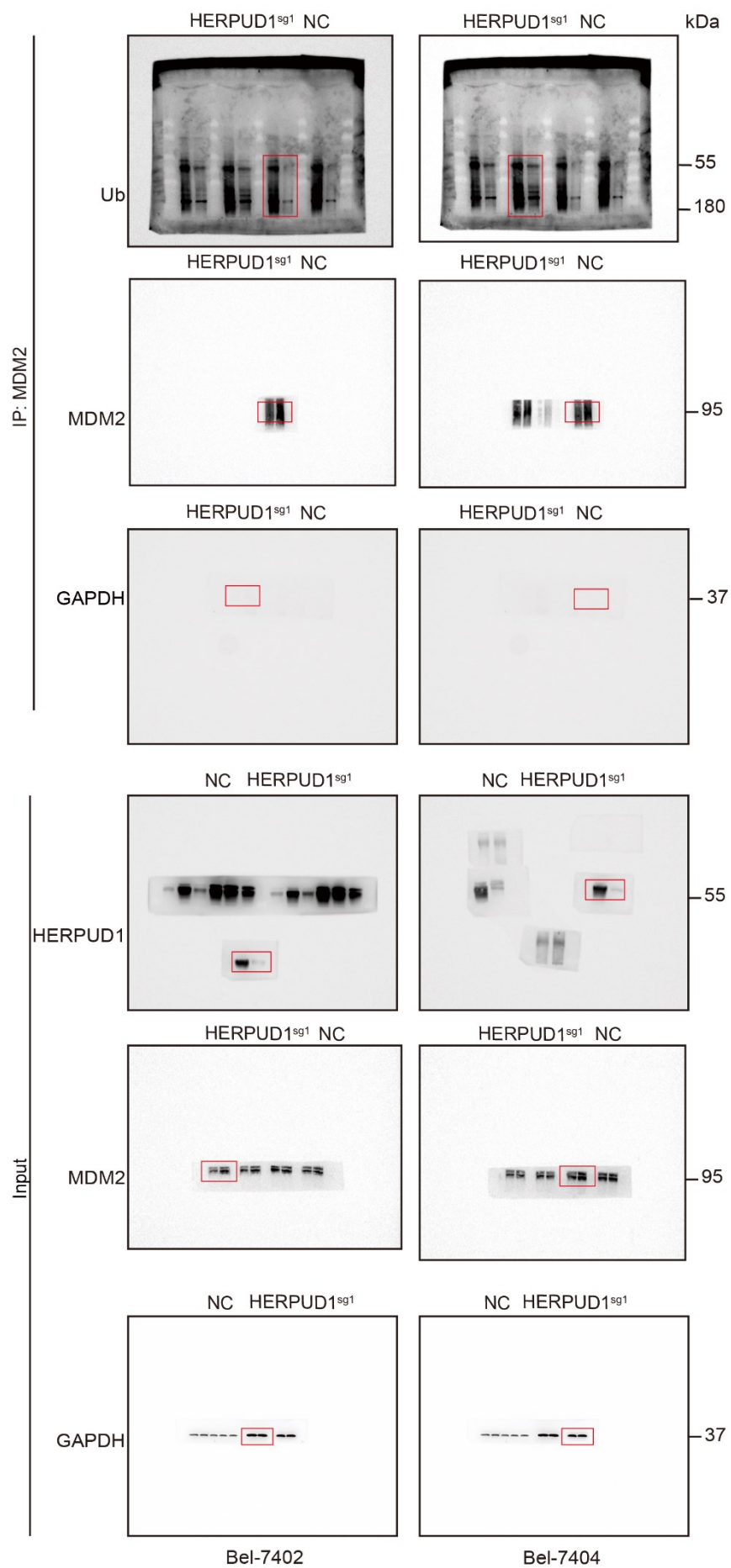

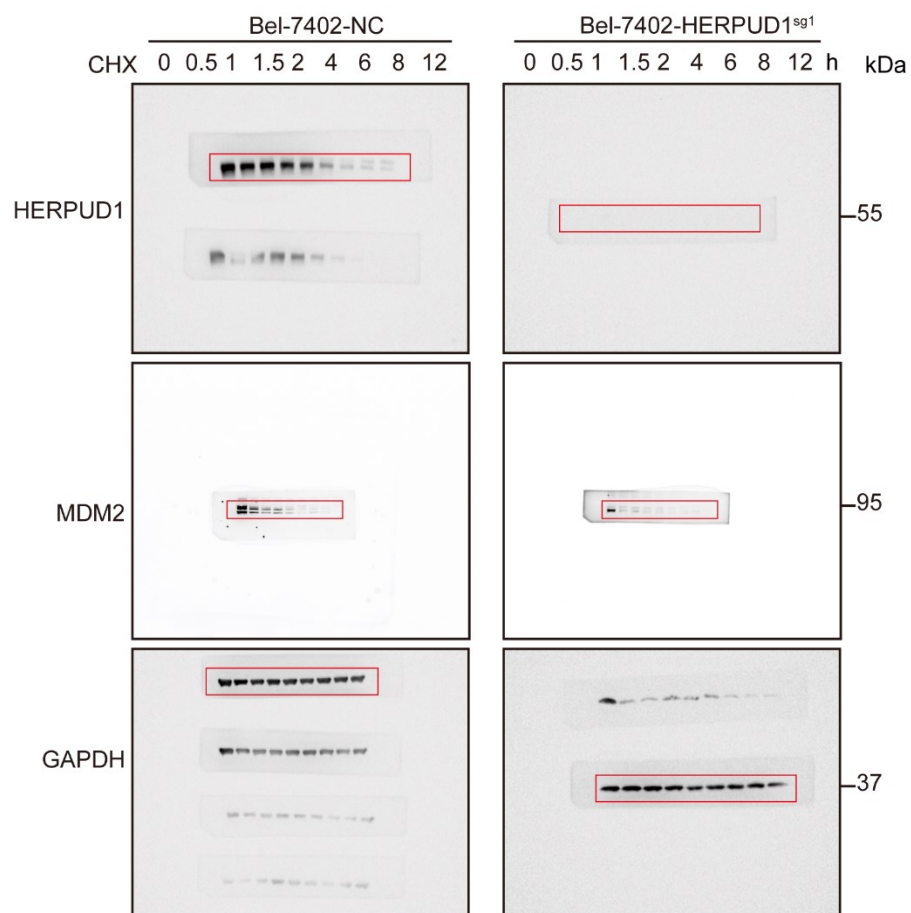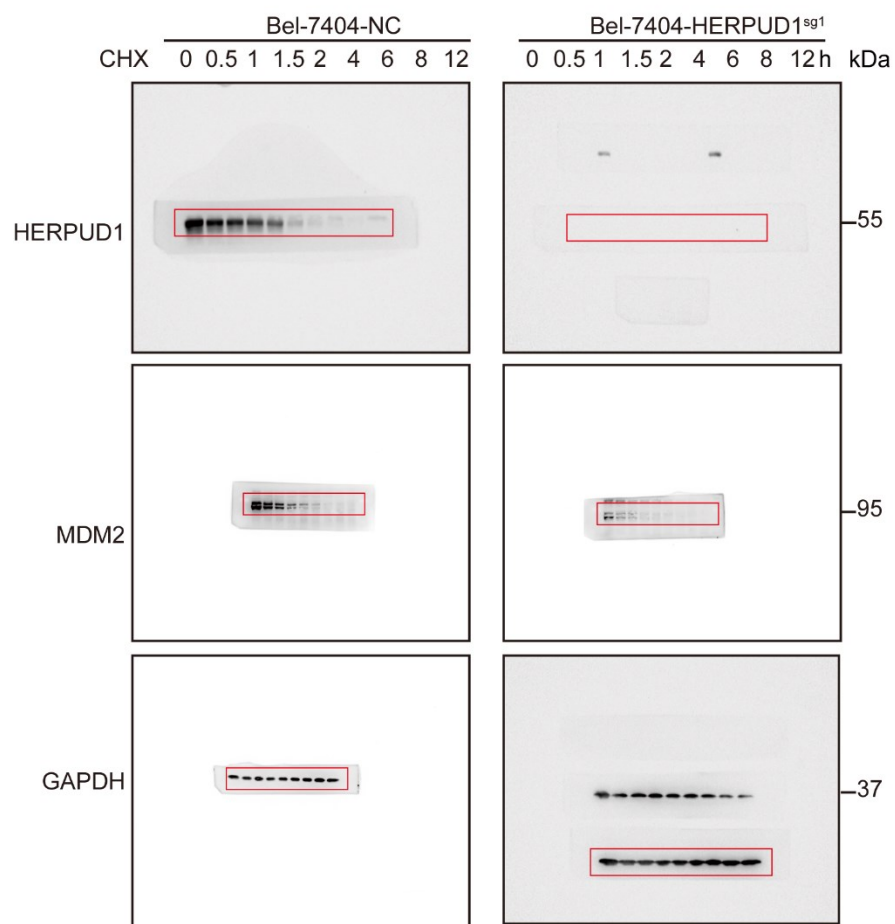

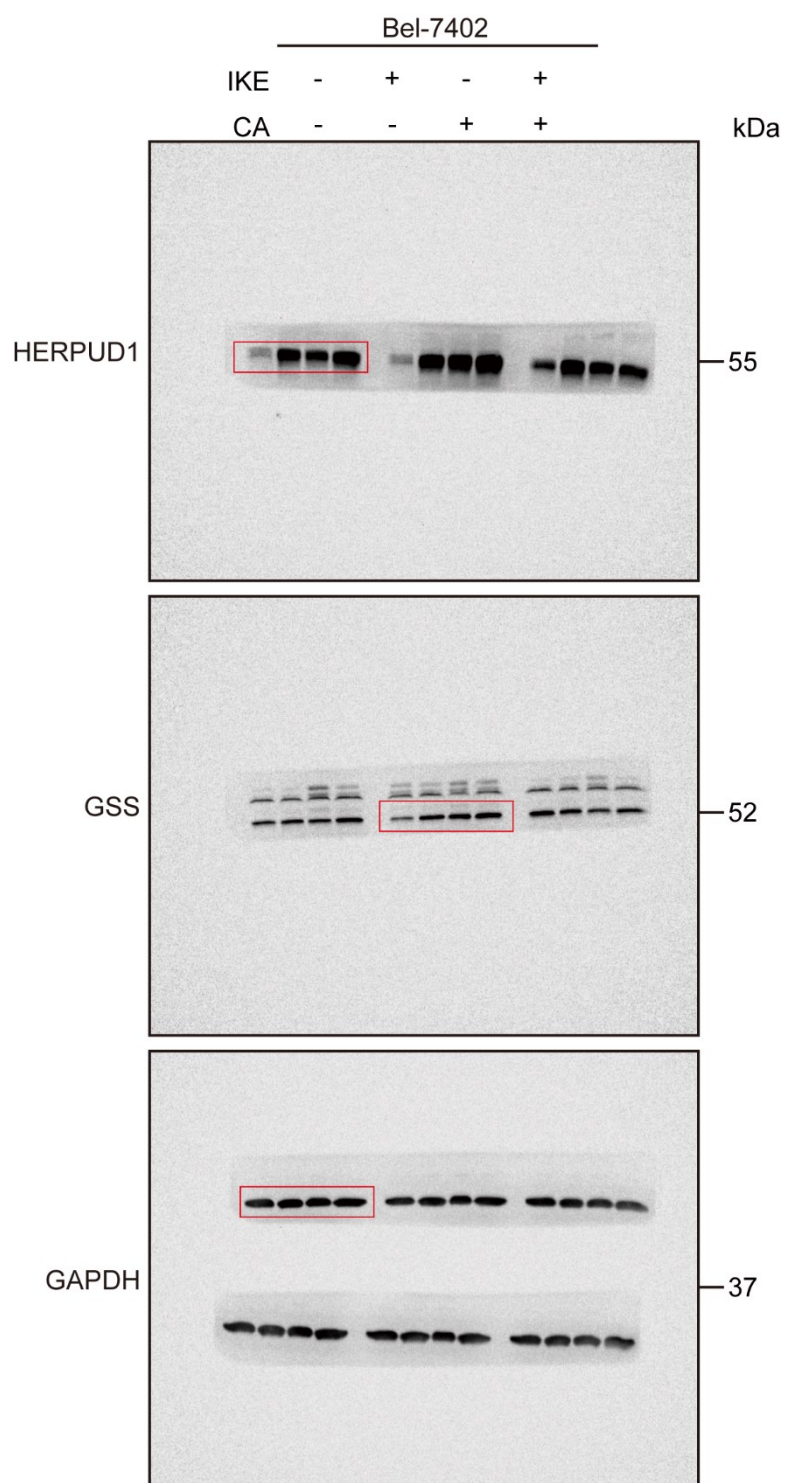

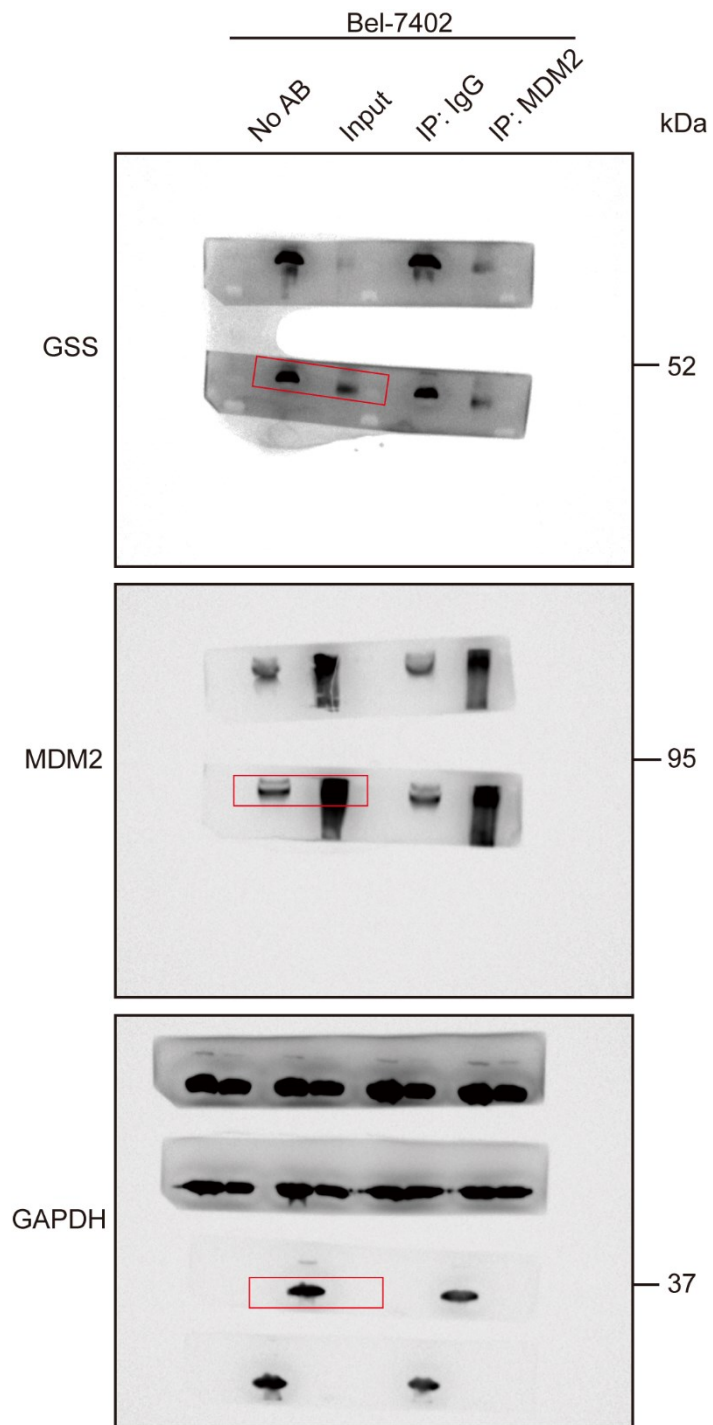

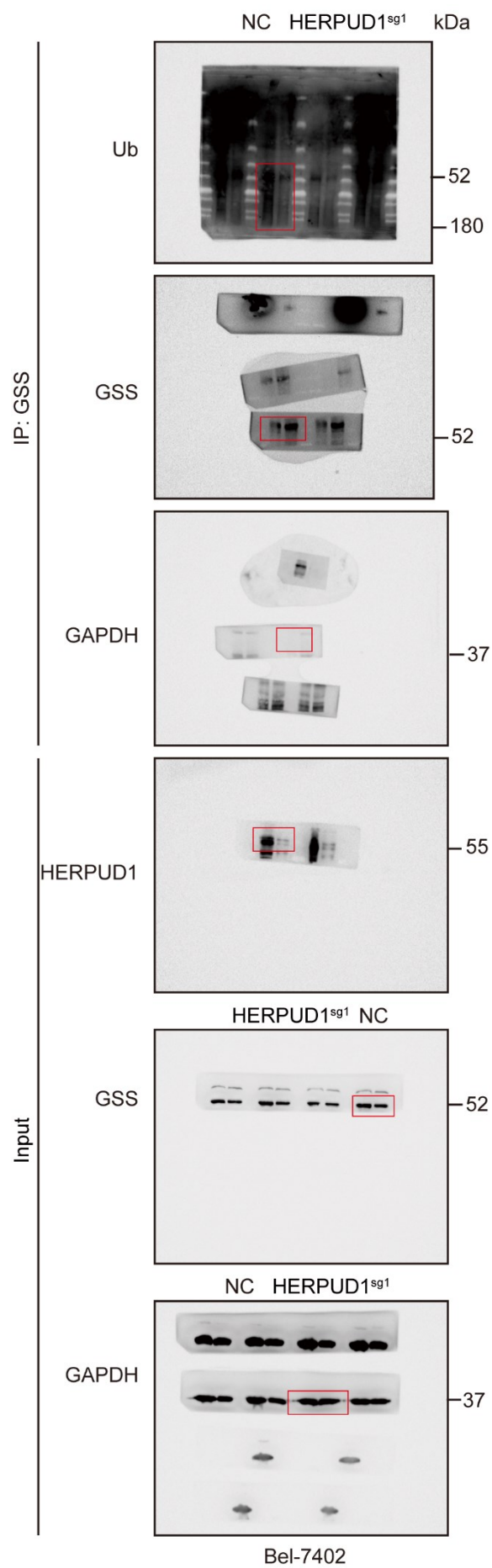

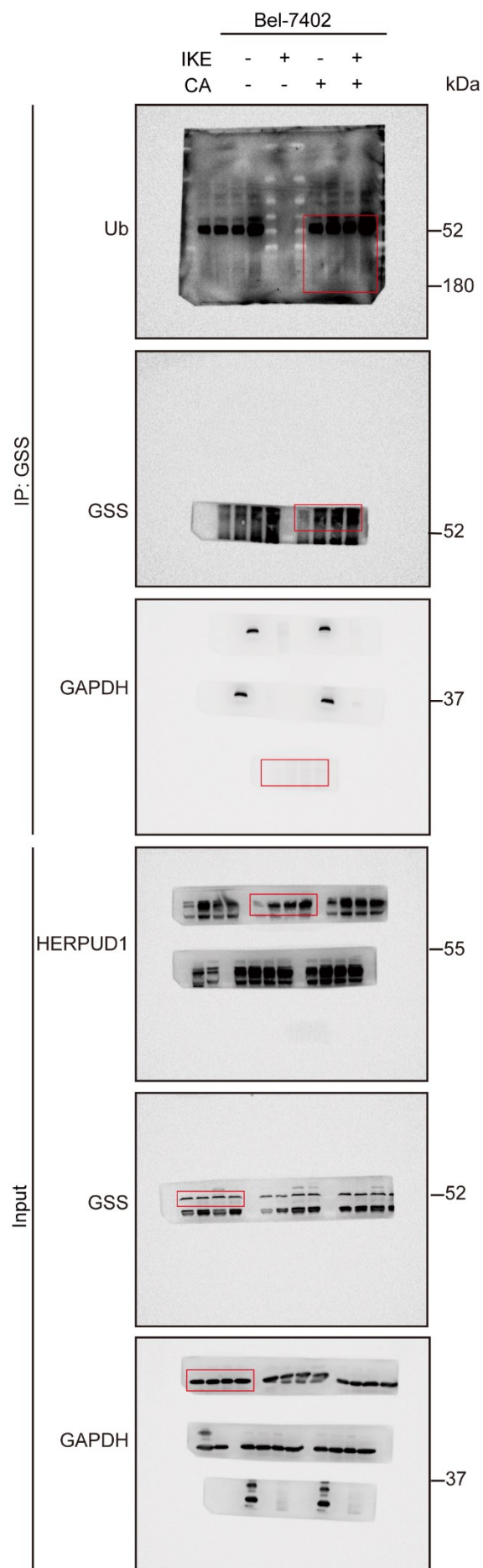

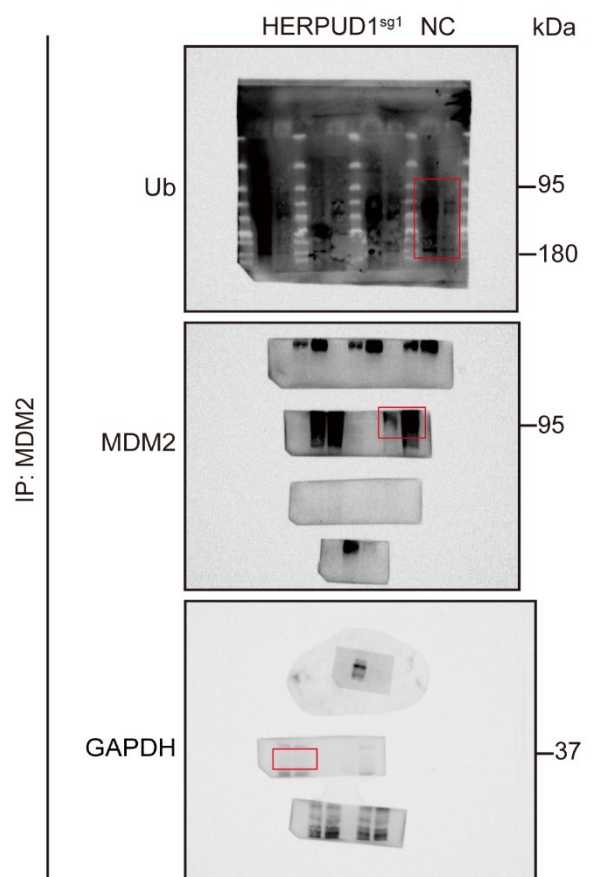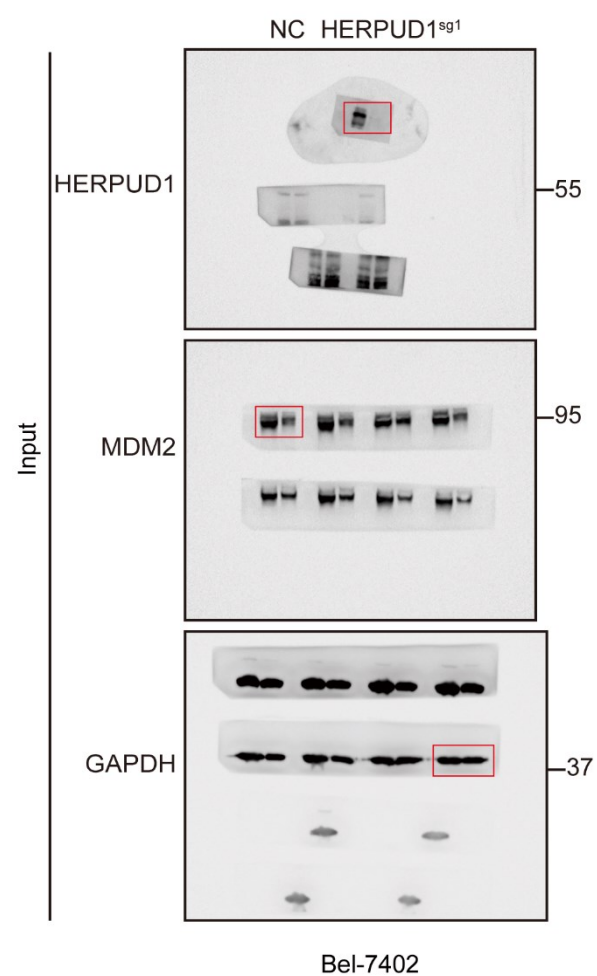

Supplement: Supplementary file 2 — Original Data File [file 41420_2022_1169_MOESM2_ESM.pdf]
